# Inherent Temporal Metamaterials with Unique Time‐Varying Stiffness and Damping

**DOI:** 10.1002/advs.202404695

**Published:** 2024-09-25

**Authors:** Zhiyuan Liu, Kaijun Yi, Haopeng Sun, Rui Zhu, Xiaoming Zhou, Gengkai Hu, Guoliang Huang

**Affiliations:** ^1^ School of Aerospace Engineering Beijing Institute of Technology Beijing 100081 P. R. China; ^2^ Department of Mechanics and Engineering Science College of Engineering Peking University Beijing 100871 P. R. China

**Keywords:** damping, signal processing, stiffness, temporal metamaterials, virtual resonators

## Abstract

Time‐varying metamaterials offer new degrees of freedom for wave manipulation and enable applications unattainable with conventional materials. In these metamaterials, the pattern of temporal inhomogeneity is crucial for effective wave control. However, existing studies have only demonstrated abrupt changes in properties within a limited range or time modulation following simple patterns. This study presents the design, construction, and characterization of a novel temporal elastic metamaterial with complex time‐varying constitutive parameters induced by self‐reconfigurable virtual resonators (VRs). These VRs, achieved by simulating the resonating behavior of mechanical resonators in digital space, function as virtualized meta‐atoms. The autonomously time‐varying VRs cause significant temporal variations in both the stiffness and loss factor of the metamaterial. By programming the time‐domain behavior of the VRs, the metamaterial's constitutive parameters can be modulated according to desired periodic or aperiodic patterns. The proposed time‐varying metamaterial has demonstrated capabilities in shaping the amplitudes and frequency spectra of waves in the time domain. This work not only facilitates the development of materials with sophisticated time‐varying properties but also opens new avenues for low‐frequency signal processing in future communication systems.

## Graphical abstract

A new type of temporal metamaterial is introduced, featuring complex, time‐varying constitutive parameters generated by self‐reconfigurable virtual resonators (VRs). By programming the dynamic responses of these VRs in the digital domain, various time‐varying patterns within the same metamaterial can be easily achieved, creating exciting possibilities for advancements in wave engineering.

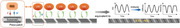

## 1 Introduction

Over the past several decades, the ability to engineer materials with exotic constants has opened new opportunities for wave manipulation.^[^ ^1^, ^2^, ^3^, ^4^, ^5^, ^6^, ^7^, ^8^, ^9^, ^10^ ^]^ To further advance the fundamental theories of classic wave‐matter interactions and enable new functionalities, time as an additional dimension has been introduced into material parameters,^[^ ^11^, ^12^ ^]^ giving rise to the emergence of temporal metamaterials.^[^ ^13^ ^]^ In these novel materials, one or more parameters are modulated over time, inducing rich exotic wave phenomena by engineering time‐domain inhomogeneities. For instance, a sudden change in material properties over a very short period creates a time interface, causing waves to reflect at this interface with frequency shifts due to momentum conservation.^[^ ^12^ ^]^ Continuous tuning of material properties in the time domain leads to entirely different phenomena. When the modulation frequency is much lower than the frequencies of the waves, adiabatic pumping^[^ ^14^, ^15^ ^]^ is achieved. Increasing the modulation frequency results in *k*‐bandgaps and parametric amplification.^[^ ^16^ ^]^ Further merging fast time‐domain modulation with space periodicity induces nonreciprocal wave propagation.^[^ ^17^ ^]^ Since wave modes are strongly controlled by the time‐varying patterns of material properties, an important yet very challenging research direction is to physically achieve complex time‐varying patterns in materials.^[^ ^12^, ^18^ ^]^

One feasible approach to change material properties in the time domain is by exploiting the coupling effect between external fields and materials. For example, the capacitance values of varactor diodes depend on the external voltages applied to them, making them basic elements for designing time‐varying transmission lines^[^ ^19^ ^]^ and metasurfaces.^[^ ^20^, ^21^, ^22^, ^23^, ^24^ ^]^ By directly tuning the time‐varying patterns of the external voltages, these transmission lines and metasurfaces exhibit different properties and functionalities. Photosensitive^[^ ^25^, ^26^ ^]^ and magnetosensitive materials^[^ ^27^, ^28^, ^29^ ^]^ have also been used to induce time‐varying properties by applying pumping pulses or magnetic fields to the materials. The properties of metamaterials are primarily influenced by the topological configurations of their microstructures. This characteristic offers an additional approach to temporally modulate material properties. Reconfiguring the shapes and sizes of metamaterials' microstructures through mechanical actuations has achieved time‐varying effects on different scales.^[^ ^14^, ^30^, ^31^ ^]^ However, these existing temporal materials or systems rely on external mechanical, electrical, or magnetic actuation to induce time‐varying effects. As a result, their properties have only been shown to change abruptly within a narrow range or to be time‐modulated in very simple patterns, significantly impeding the exploration of new phenomena and functionalities arising from complex time‐varying patterns.

Time‐varying metamaterials fall under the category of tunable metamaterials. In terms of tunability and reconfigurability, piezoelectric metamaterials, whose properties are primarily determined by external shunts, can achieve widely tunable effective parameters.^[^ ^32^, ^33^, ^34^, ^35^ ^]^ By continuously switching the shunts in the time domain, the properties of piezoelectric metamaterials have also been shown to be temporally variable.^[^ ^16^, ^17^ ^]^ Nevertheless, there have been no significant breakthroughs in the range of time‐varying property adjustments and the complexity of varying patterns. Recently, analog shunts have been replaced by digital circuits in the design of advanced piezoelectric metamaterials.^[^ ^17^, ^36^, ^37^, ^38^ ^]^ The properties and functionalities of these metamaterials are induced by transfer functions (TFs) operating within digital circuits, highlighting a potential direction for achieving more flexible tuning of material parameters, including within the time domain.

**Figure 1:**
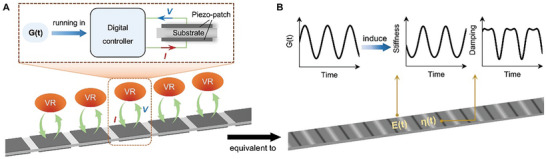
Design concept of temporal elastic metamaterials. A) Schematic diagram of an elastic metamaterial with time‐varying VRs. The VR is represented by a transfer function (TF) *G*(*t*), it is running in a digital controller, its resonance behaviors can autonomously vary in time domain. The VRs are coupled to elastic substrates through piezoelectric materials to construct metamaterials. B) The effective stiffness and damping characteristics of the metamaterials with VRs become time‐varying induced by the time‐dependent TF.

Here, we propose a method to design temporal metamaterials with highly tunable time‐varying constitutive properties by harnessing built‐in self‐reconfigurable meta‐atoms. The meta‐atoms are realized by mimicking dynamic behavior of mechanical resonators (MRs) in digital space, namely, a sort of virtual resonators (VRs) are obtained. These VRs are mathematically represented by TFs (denoted as *G*) and running in digital controllers, with voltage (*V*) as input and current (*I*) as output (see the upper figure in **Figure** **1**A). The VRs are coupled to elastic substrates through piezoelectric materials to construct metamaterials (a continuous beam is used as the substrate in this work, as schematically illustrated by the lower figure in Figure 1A). Due to the virtual nature, VRs’ dynamic properties, including number of resonating degrees of freedom (DOFs), resonance frequencies and resonating strengths, can autonomously vary over time by using time‐dependent TFs. As a result, mechanical properties of the constructed metamaterials, including stiffness and damping, become inherently time‐varying induced by the VRs (Figure 1B). Following the above method, we have designed and fabricated a metabeam. Our theoretical, numerical, and experimental results show that the temporal variation patterns in the metabeam are solely governed by the parameters of the VRs. By programming the dynamic behavior of the VRs in the time domain, these patterns can be adjusted to achieve desired periodic or aperiodic forms. This capability has proven useful in temporally shaping the amplitudes and frequency spectra of low‐frequency elastic waves.

## 2 Results and Discussion

### 2.1 Transfer Functions for Simulating Virtual Resonators

**Figure 2:**
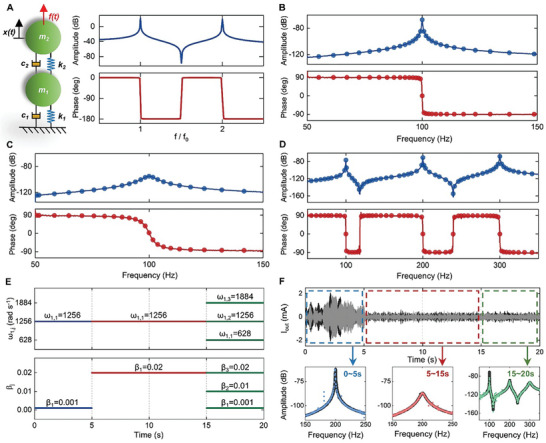
Resonant behaviors of MRs and VRs. A) A two DOFs MR represented by a lumped model and the graphical illustrations of the driving‐point admittance *H*(ω) of the model, *f*_0_ is the first resonance frequency. Expression of the admittance is given in Equation S1 (Supporting Information), *m*_1_ = *m*_2_  =  1, *c*_1_ = *c*_2_  =  2, *k*_1_ = *k*_2_  = 10^6^ . B) Resonant behaviors of a VR designed to mimic a MR with one resonance at 100 Hz. Solid lines are results of experiments, solid points are analytical results. C) A VR with less strong resonating strength than that in (B). D) Resonant behaviors of a VR designed to mimic MRs with three resonances at 100, 200, and 300 Hz. E) The upper and lower panels show the time‐varying patterns of the parameters ω_1,*j*_ and β_*j*_ in the designed time‐varying TF, respectively. F) The upper panel is the measured time‐domain current signal generated by the time‐varying TF, the black and gray lines are the numerical and experimental results, respectively. The lower panel shows the spectra of the measured signal between different time intervals, the black solid lines are the numerical results, the dotted lines are the experimental results.

The key to realizing our temporal metamaterials is to design the VR. To understand how it is obtained, first, we review the fundamental characteristics of MRs. Without loss any genericity, we use a two DOFs resonator shown in **Figure** **2**A represented by a lumped model for interpretation. A harmonic driving force *f* (*t*) = *F*sin(ω*t*) is applied at the second mass, the corresponding response of it can be expressed as *x* (*t*) =  *X*sin(ω*t* + φ), here, φ is a constant phase. A graphical illustration of the driving‐point admittance (which is *H*(ω) = *X*(ω)/*F*(ω) , details are in Note S1, Supporting Information) is shown in Figure 2A, it is observed that the dynamic behaviors of a MR are characterized by resonances, anti‐resonances and the accompanying phase inversion phenomena. Therefore, if we can mimic the above resonating behaviors in the digital space, a VR is realized.

According to Foster's theorem,^[^ ^39^ ^]^ an electric system with multiple resonances and anti‐resonances can be achieved by assigning poles and zeros. Inspired by this, the TF to realize a VR is given by

(1) $$\begin{array}{c} G=i\alpha \omega \frac{\prod_{j\;=\;1}^{N}\left(-\omega^{2}+i\beta_{j}\omega_{2,j}\omega +\omega_{2,j}^{2}\right)-\prod_{j\;=\;1}^{N}\left(-\omega^{2}+i\beta_{j}\omega_{1,j}\omega +\omega_{1,j}^{2}\right)}{\prod_{j\;=\;1}^{N}\left(-\omega^{2}+i\beta_{j}\omega_{1,j}\omega +\omega_{1,j}^{2}\right)} \end{array}$$

The above function involves several parameters, α is a factor, proportionally changes the amplitude of the responses; *N* is the number of resonance; ω_1,*j*_ and β_*j*_ together determine the resonance frequencies according to $\omega_{1,j}\sqrt{1-\beta_{j}^{2}/4}$, if the values of β_*j*_ are small, the resonance frequencies approximately are ω_1,*j*_; β_*j*_ determines the resonating strength of the *j^th^* resonance. ω_2,*j*_ determines the *j^th^* anti‐resonance frequency, it is constrained by ω_1,*j*_ < ω_2,*j* + 1_ < ω_1,*j* + 1_ due to the requirement of stability.

The TF given in Equation (1) is based on Foster's theorem but has been significantly modified. We first have multiplied the circular frequency ω in front of Foster's formula to add a zero at the origin to remove the static response of the VR, this process makes the VR more robust when it is simulated in a digital controller in real time. Second, the highest‐order term of ω in the numerator has been eliminated by a subtraction operation to make the VR causal. Third, we have used complex poles and zeros instead of real ones, this gives us freedom to control the resonating strengths of the VR. Lastly, time‐varying parameters of the TF are used to make the resonating behaviors automatically change in time domain.

The proposed VR not only can mimic the resonant behavior of the 2‐DOFs MR in Figure 2A (Note S1, Supporting Information), but also it can be tuned to achieve arbitrary resonant responses. This merit is demonstrated through analytical and experimental studies. The analytical frequency response function (FRF) of the VR can be obtained through Equation (1). During the experiments, the VR is operating in a digital circuit, a signal generator is used to input voltage into the VR, and a data acquisition instrument is used to measure the output current (Note S2, Supporting Information). Using the input and output signals, we can calculate the FRF of the VR. In Figure 2B, we chose the following parameters *N*  =  1, ω_1,1_ =  628 and β_1_ =  0.001 to mimic a MR with one resonance at 100 Hz. Next, we prefer a less strong resonance, with a resonating amplitude only half of that in Figure 2B, for this end, we increase β_1_ of the TF to be 0.02, the resonance behavior is exactly tailored to the desired profile, as illustrated in Figure 2C. In another case, we would like to generate a resonator with 3 resonances at frequencies 100, 200, and 300 Hz, therefor, parameters of the TF are chosen as *N*  =  3, ω_1,j  =  1, 2, 3_ =  628,  1256,  1884, and β_j  =  1, 2, 3_ =  0.001, when this new TF is downloaded into the controller, we observe 3 resonances just at the desired locations (Figure 2D). These results clearly verify that the number of DOFs, resonance frequencies and resonating strengths of the VR can be freely and precisely customized by tuning the TF.

We now examine the time‐varying characteristics of the VR. Since the resonant properties of the VR are simply determined by parameters of the TF. Therefore, if these parameters are automatically varying in time domain, namely, using a time‐variant TF, we can realize a resonator whose behaviors will dynamically change in a desired temporal pattern. As an example for demonstration, we expect the VR to resonate at 200 Hz for 5 s, then the resonating strength deceases to a half and last for 10s, after that, the VR simultaneously resonates at 3 different frequencies (100, 200 and 300 Hz). To achieve such complicated time‐varying pattern, parameters of TF are designed to vary in time domain in a way illustrated in Figure 2E. Figure 2F shows the time‐domain current signal generated by the time‐variant TF, the spectra of the signals between different time intervals are also shown. It is clearly observed that the resonating properties of the VR automatically vary in time domain just as designed.

### 2.2 Inherent Time‐Varying Stiffness and Damping

To analyze the time‐varying constitutive parameters, the metabeam in Figure 1A is studied. Since a deformed piezo‐patch is equivalent to a voltage source (*V*_s_) plus a capacitor ($C_{p}^{s}$) in series (Note S3, Supporting Information), the TF for simulating a VR connected to a patch is modified to

(2) $$G^{*}=i\omega C_{p}^{s}\frac{\prod_{j\;=\;1}^{N}\left(-\omega^{2}+i\beta_{j}\omega_{2,j}\omega +\omega_{2,j}^{2}\right)-\prod_{j\;=\;1}^{N}\left(-\omega^{2}+i\beta_{j}\omega_{1,j}\omega +\omega_{1,j}^{2}\right)}{\frac{1}{1-k_{31}^{2}}\prod_{j\;=\;1}^{N}\left(-\omega^{2}+i\beta_{j}\omega_{1,j}\omega +\omega_{1,j}^{2}\right)-\prod_{j\;=\;1}^{N}\left(-\omega^{2}+i\beta_{j}\omega_{2,j}\omega +\omega_{2,j}^{2}\right)}$$

here, *k*_31_ is the extensional coupling factor of the piezo‐patch. The values of ω_1,*j*_ and ω_2,*j*_ must meet the constrain $\omega_{1,j}\leq \omega_{2,j}\leq {\left(\frac{1}{1-k_{31}^{2}}\right)}^{\frac{1}{2N}}\omega_{1,j}$ for the stability of the modified TF.

**Figure 3:**
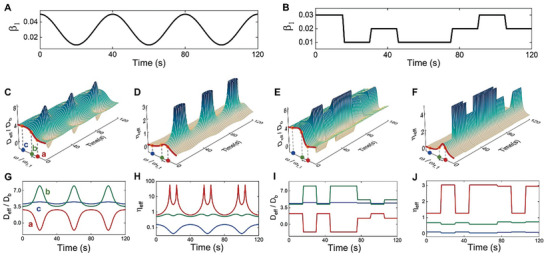
Time‐varying stiffness and damping of a metabeam. A,B) Periodical and aperiodical time‐varying patterns of parameter β_1_ of a one‐pole TF. C–F) The time‐varying effective bending stiffness (*D*_eff_) and loss factor (η_eff_) of the metabeam induced by the temporal β_1_ in Figure 3A (C,D) and in Figure 3B (E,F). The effective bending stiffness is normalized using *D*_eff_/*D_b_*, *D*_b_ is the bending stiffness of the bare beam. G–J) Details of the time‐varying constitutive parameters at frequencies a, b, and c. Frequencies a, b are close to the resonance frequency ω_1,1_ of the VR, frequency c is away from it, as illustrated in Figure 3C.

**Figure 4:**
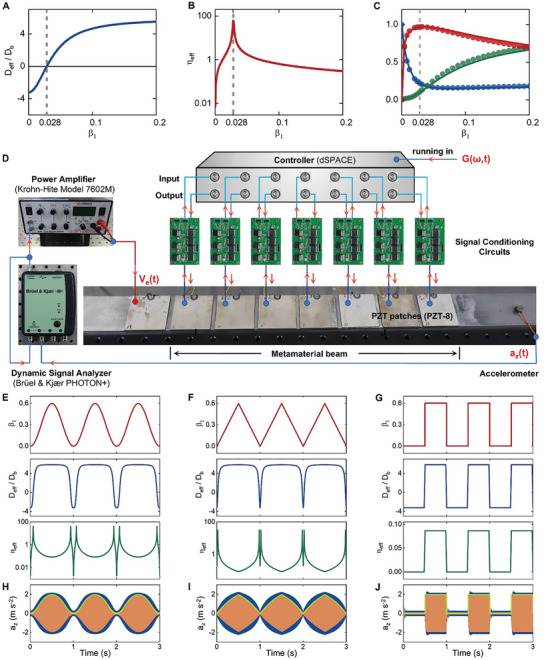
Demonstration of the time‐varying stiffness and damping. Variation of A) the effective bending stiffness and B) loss factor of the metabeam by tuning β_1_ when a one‐pole TF is used. At β_1_ =  0.028, the bending stiffness is zero, the loss factor has an extremum. C) The reflection (blue), transmission (green) and absorption (red) coefficients of the metabeam corresponding to different constitutive parameters. Solid lines are theoretical results, solid dots are calculated by using a fully coupled numerical model. D) The fabricated metabeam with seven unit cells and the experimental setting up for verifying its time‐varying properties. On the left side of the metabeam, an additional pair of piezo‐patch is used for generating incident waves. On the right side, an accelerometer is used to measure the transmitted waves. E–G) Upper panels: the parameter β_1_ of a one‐pole TF (the resonance frequency is 633 Hz) is modulated in time domain according to a E) sine, F) trigonometric and G) square function, respectively. The modulation frequencies are all 1 Hz; central and lower panels: the induced time‐varying bending stiffness and loss factor at 633 Hz. H–J) the amplitudes of the transmitted waves corresponding to H) the sine, I) trigonometric, and J) square modulation of β_1_ in time domain, respectively. H–J) The frequencies of the incident waves are all 633 Hz. The blue and orange solid lines are the results of experiments and simulations using the fully couple model, respectively. The green solid lines are results obtained using the homogenized metabeam model.

We consider the flexural motion of the metabeam, when it is characterized as a homogenized solid, the associated constitutive parameters are the effective bending stiffness (*D*_eff_) and loss factor (η_eff_). To obtain the two effective parameters, we assume the metabeam works in the adiabatic time‐varying region,^[^ ^40^ ^]^ namely, the motions of the materials are much faster compared with the time‐modulation speed of the materials’ parameters (therefore, the Cauchy elasticity theory is applicable); also we assume the length of the unit cell is much shorter than the wavelength. Under these assumptions and employing the classic Euler‐Bernoulli beam theory, effective parameters of the homogenized metabeam are expressed as functions of ω_1,*j*_,  β_*j*_ (Experimental Section), they can be significantly modified by changing parameters of VR (Note S4, Supporting Information). Therefore, by temporally tuning one or several of these parameters, we can freely modulate the effective constitutive parameters. For example, when a one‐pole TF is used, we periodically (**Figure** **3**A) or aperiodically (Figure 3B) tune β_1_ of the TF in time domain, the resultant time‐varying effective bending stiffness and loss factor are illustrated in Figure 3C–F, respectively. The constitutive parameters at frequencies near the resonance frequency ω_1,1_ (see Figure 3G–J, for more details) are dramatically modulated. The effective bending stiffness can be modulated within the positive region (for examples, at frequencies b and c in Figure 3G,I) or spanning from negative to positive regions (at frequency a in Figure 3G,I as an example). In some cases, the loss factor shows recurring extremums (see frequency a in Figure 3H), they occur when the bending stiffness across zero, this feature is more clearly revealed in **Figure** **4**A,B. We note that the effective constitutive parameters can also be time‐modulated by varying ω_1,1_ of the one‐pole TF; furthermore, by using multi‐pole TFs, we can simultaneously modulate the constitutive parameters within several frequency bands (Figures S5–S7, Supporting Information).

### 2.3 Experimental Demonstration of the Time‐Varying Constitutive Parameters

The time‐varying constitutive parameters are difficult to be directly measured, here, we indirectly verify the existing of them by studying the wave transmission properties of the metabeam. First of all, to understand the underlying mechanism of this method, we analyze the influences of the constitutive parameters on the wave transmission. We consider a metabeam with 7 unit cells and use a one‐pole TF to simulate the VR. A monochromatic wave at ω_1,1_ is incident from the left side of the beam. We continuously vary β_1_ to change the effective bending stiffness and loss factor of the beam at ω_1,1_ (see Figure 4A,B) and calculate the corresponding reflection, absorption, and transmission coefficients, the results are illustrated in Figure 4C, the solid lines are obtained using the effective parameters shown in Figure 4A,B (The method is introduced in Note S6, Supporting Information), and the discrete points are results obtained using a fully coupled metabeam model (Figure S10, Supporting Information). The results obtained using the homogenized and fully coupled models are well coincident with each other, which verify that tuning the value of β_1_ actually changes the effective parameters of the metabeam. It is also observed from Figure 4A–C that the variation of the constitutive parameters is clearly reflected on the changing of the amplitude of the transmitted wave (it can be further understood according to the transfer matrix method in Note S8, Supporting Information). For a small β_1_, the effective bending stiffness has a very large negative value, most of the incident wave is reflected, the transmission is negligible. When β_1_ increases from 0 to 0.028, the effective bending stiffness changes from negative to zero and the effective loss factor also increases to the maximum. As a result, the reflection caused by the negative stiffness reduces fast, the dissipation caused by the damping increases dramatically. Nevertheless, the sum of the reflected and dissipated wave energy decreases. Consequently, the amplitude of the transmitted wave increases when β_1_ enlarges in this region. As β_1_ further increases from 0.028 to a larger value, the bending stiffness grows and the loss fact drops, the reflection remains almost unchanged and the dissipation weakens, therefore, the transmitted wave increases. Using the above revealed correlation between the constitutive parameters and transmission, we can indirectly observe the time‐varying constitutive parameters of the metabeam by measuring the amplitudes of the transmitted waves in time domain through experiments.

The fabricated metabeam used in the experiments is illustrated in Figure 4D. A one‐pole TF with fixed resonance frequency at 633 Hz is used to simulate the VR. We modulate the value of β_1_ in time domain to induce time‐varying constitutive parameters. Sinusoidal, trigonometric and square functions are used for demonstration in the experiments, as illustrated in the upper panels of Figure 4E–G, the modulation frequency is 1 Hz. The central and lower panels in Figure 4E–G show the corresponding theoretical time‐varying constitutive parameters of the beam, they span several orders of magnitudes. These time‐varying parameters make the amplitudes of waves transmitted through the beam dramatically changes, as verified by the results (the green solid lines in Figure 4H–J, the frequency of the incident wave is 633 Hz) obtained using the homogenized metabeam model (the method is introduced in Note S6, Supporting Information). To study the wave transmission in experiments, we use an additional pair of patches on the left side of the metabeam to generate a monochromatic incident wave at 633 Hz with a constant amplitude. The transmitted waves corresponding to different modulation patterns are measured, as shown in Figure 4H–J. We can see that the amplitudes of the transmitted waves are continuously modulated in time domain just follow the predicted patterns using the homogenized metabeam model, which indirectly demonstrate the existence of time‐varying constitutive parameters. The stiffness of our metamaterial exhibits temporal variability spanning a wide range, with a variation exceeding eight times the initial stiffness. Additionally, the damping capability undergoes continuous temporal modulation, with the differential between its maximum and minimum values exceeding several orders of magnitude. We also show the numerical results obtained by using the fully coupled model (Experimental Section) in Figure 4H–J, they are in good agreements with the experimental results, which further verify the accuracy of our experiments (a comprehensive error analysis is presented in Note S9, Supporting Information).

### 2.4 On‐Demand Tuning of the Time‐Varying Constitutive Parameters

**Figure 5:**
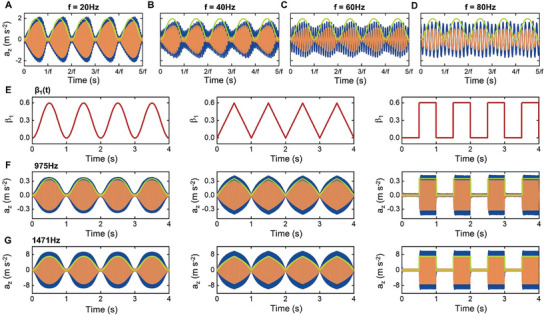
On‐demand tuning of the time‐varying constitutive parameters. A–D) Variation of the transmitted waves through the metabeam when the modulation frequencies are A) 20 Hz, B) 40 Hz, C) 60 Hz and D) 80 Hz, respectively. β_1_ is modulated in a sinusoidal pattern. E) Parameter β_1_ of a one‐pole TF is modulated in the time domain according to a sine, trigonometric and square function. F,G) Transmitted waves through the metabeam when resonance frequency of the VR is F) 975 Hz or G) 1471 Hz. Frequency of the incident wave is equal to the VR's resonance frequency in each case. The blue and orange lines are the results of experiments and simulations, respectively. The green solid lines are the results obtained using theoretical effective constitutive parameters.

The virtual nature of the VRs enables us to easily tune the time‐varying stiffness and loss factor of the metabeam on‐demand. Results in Figure 4H–J already indicate that the modulation pattern of the constitutive parameters can be flexibility tuned to any desired fashion by programming the VRs. Next, we demonstrate that the modulation frequency of the constitutive parameters can be tuned within a certain range. We take the sinusoidal modulation pattern as an example and measure the transmitted waves for different modulation frequencies, the results are shown in **Figure** **5**. Frequencies of the incident waves are all 633 Hz in these cases. When the modulation frequency is less than 60 Hz (about 0.1 times of the response frequency of the material, see Figure 5A–C), the amplitudes of the transmitted waves in the experiments and simulations approximately follow the patterns predicted by the homogenized model, which means the adiabatic assumption is fulfilled, the metabeam can be treated as a temporal Cauchy‐elastic medium with strongly time‐modulated constitutive parameters. For higher modulation frequencies (e.g., 80 Hz in Figure 5D), the metabeam is stable, but the adiabatically homogenized model based on Cauchy's theory is no longer suitable to describe the dynamic responses of it, advanced theories considering nonlocal effects in the time dimension are needed.^[^ ^40^ ^]^ Therefore, the modulation frequency should be less than 0.1 time of the wave frequency in order to modulate the waves in desired manners. This conclusion is further verified by the results in Figure S18 (Supporting Information).

We also demonstrate that time‐varying constitutive parameters can be achieved at desired frequencies by tuning the resonance frequencies of VRs. In the above studies, the resonance frequency of the VR is fixed to be 633 Hz, now we change it to 975 or 1471 Hz. By modulating parameter β_1_ (the patterns are shown in Figure 5E), we can achieve remarkable time‐varying constitutive parameters near 975 (see Figure 5F) or 1471 Hz (see Figure 5G). The maximum resonance frequency is constrained by the length of the unit cell of the metabeam, as we have assumed during homogenization that the wavelength of the flexural wave in the beam is much longer than the length of a single unit cell. For the beam shown in Figure 4D, the maximum resonance frequency should be less than 1500 Hz (details are provided in Note S10, Supporting Information). Decreasing the length of the unit cell can increase the maximum resonance frequency.

### 2.5 Mechanism of Generating Time‐Varying Properties Based on Virtual Resonators

**Figure 6:**
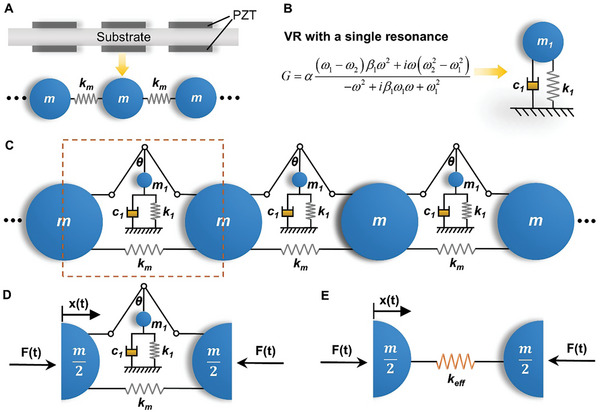
A discrete model of the metabeam. A) A discrete mass‐spring chain model to represent the substrate beam with short‐circuited piezo‐patches. B) A one‐pole VR can be replaced by a MR with the equivalent resonant behavior. C) Coupling the resonators in (B) to the chain in (A). D) A unit cell of the discrete metamaterial. E) The equivalent model of the unit cell.

In this section, we use a discrete model to explain the mechanism of generating time‐varying constitutive parameters based on virtual resonators. We consider longitudinal motion in the metabeam. The substrate beam with short‐circuited piezo‐patches can be discretely represented by a chain composed of mass *m* and spring *k*_m_, as shown in **Figure** **6**A. The VR mimics behavior of a MR. Therefore, we can also use a spring‐mass lumped model to replace it. For a one‐pole VR, the lumped model with the equivalent resonant behavior is shown in Figure 6B. In the continuous metabeam, VRs are coupled to the beam through electromechanical coupling effects of the piezo‐patches. Such effects are introduced into the discrete model through rigid bars and hinges, as illustrated in Figure 6C. Angle between a rigid bar and the vertical direction is θ, the tangent value of it is equal to the electromechanical coupling factor γ of a piezo‐patch (Equation S26, Supporting Information). The two rigid bars are connected with each other and to the masses through hinges. The theory for this equivalence is provided in Supplementary Note 11.

A unit cell of the discrete model is shown in Figure 6D. *F*(*t*) represents the internal force on the mass. *x*(*t*) is the displacement of the left mass. The equilibrium equation of the left mass is

(3) $$F+\frac{1}{2}m\omega^{2}x-2k_{m}x-2\mathrm{ta}n^{2}\theta \left(-m_{1}\omega^{2}+i\omega c_{1}+k_{1}\right)x=0$$

Next, we equate the unit cell to a model with effective complex stiffness *k*_eff_ (Figure 6E). The equilibrium equation of the effective model is $F+\frac{1}{2}m\omega^{2}x-2k_{\mathrm{eff}}x\;=\;0$. By comparing it with Equation (3), we derive the expressions for the effective complex stiffness and damping, which are

(4) $$\begin{array}{ccc} & & k_{\mathrm{eff}}=k_{m}\;+\tan^{2}\theta k_{1}\left(1-\frac{\omega^{2}}{\omega_{1}^{2}}+2i\omega \omega_{1}\xi \right)\\ & & \eta_{\mathrm{eff}}=\frac{\mathrm{Imag}\left(k_{\mathrm{eff}}\right)}{\left|\mathrm{Real}\left(k_{\mathrm{eff}}\right)\right|}\;=\frac{2\tan^{2}\theta k_{1}\omega \omega_{1}\xi }{\left|k_{m}+\tan^{2}\theta k_{1}\left(1-\frac{\omega^{2}}{\omega_{1}^{2}}\right)\right|} \end{array}$$

in which, $\omega_{1}=\sqrt{k_{1}/m_{1}}$ and ξ  = *c*_1_/2*m*_1_ .

From Equation (4), it is evident that if the resonance frequency ω_1_ or the damping ξ of the internal resonator varies over time, the effective stiffness and damping of the metamaterial will also become time‐dependent.

### 2.6 Discussion on Potential Applications

Amplitude, phase, and frequency modulation devices, along with various other signal processing components, are essential elements in modern communication systems.^[^ ^23^, ^24^, ^41^ ^]^ Elastic waves, compared to electromagnetic waves of the same frequency, have much shorter wavelengths and slower velocities. This characteristic provides significant advantages in terms of size, robustness, and functionality when using elastic wave‐based devices for signal processing, exemplified by the success of SAW (surface acoustic wave) filters.^[^ ^42^ ^]^ The increasing interest in using extremely low‐frequency electromagnetic waves for communication makes elastic wave‐based devices even more critical for next‐generation communication systems. Low‐frequency electromagnetic waves have long wavelengths (e.g., at 1 kHz, the wavelength is 3 × 10^5^ m), allowing them to travel farther and be less affected by the Earth's curvature compared to microwaves. They can also penetrate deeply into water and ground. However, directly processing such low‐frequency electromagnetic waves requires devices of incredibly large sizes.

In contrast, the wavelengths of comparable elastic waves are much shorter (for example, at 1 kHz, the wavelength of the flexural wave in our metabeam is only 0.8 m), making them easier to be modulated in solids. From this perspective, the proposed time‐varying metabeam has potential applications in processing low‐frequency signals. Results in Figure 4C,E–J indicate that, when the adiabatic assumption is fulfilled, the variation patterns of the amplitudes of the transmitted waves through the metabeam are nearly linearly correlated with those of β_1_ (which determines the resonating strength of the VR). By exploiting this characteristic, we can precisely shape the amplitudes of the transmitted waves. Figures 4 and 5 demonstrate that the metabeam can modulate the amplitudes of transmitted signals in a desired periodic fashion. Additionally, Figures S21 and S22 (Supporting Information) further showcases the metabeam's capacity to modulate signal amplitudes in aperiodic ways. Furthermore, the metabeam can function as a frequency‐hopping filter. The operating frequencies of such a filter automatically change in the time domain to shape the frequency spectrum of a signal sequence (Figure S23, Supporting Information), providing another additional degree of freedom in low‐frequency signal processing.

## 3 Conclusion

In summary, utilizing self‐reconfigurable built‐in meta‐atoms, we have proposed and demonstrated a novel method for designing inherent temporal metamaterials with exceptional time‐modulated stiffness and damping properties. The stiffness can continuously vary over time within a positive and negative range, with a variation exceeding eight times the original stiffness. The damping can also vary continuously in the time domain, with the difference between the maximum and minimum values exceeding a factor of hundreds. The temporal variation patterns of material parameters can be of any periodic or non‐periodic form, allowing for very flexible adjustment, and can even be operated remotely. Leveraging these merits, the proposed temporal metamaterials have potential applications in modulating the amplitudes and frequency spectra of low‐frequency waves. In addition to these remarkable functionalities, the metamaterial may serve as a platform to study temporal pumping,^[^ ^14^, ^15^ ^]^ frequency conversion at time interfaces,^[^ ^12^ ^]^ *k*‐space bandgaps,^[^ ^16^ ^]^ etc., potentially inspiring other new wave‐based devices for signal processing.

## 4 Experimental Section

### Sample Fabrication

The host beam (aluminum) in Figure 4D is manufactured using laser‐cut technique. Seven pairs of piezoelectric patches (PZT‐8) are periodically glued onto the surfaces of the host beam through epoxy. The geometry and material parameters of a unit cell are listed in Note S13, Supporting Information. The poling directions of the two patches in a unit cell are in the opposite direction. The electrodes are on the upper and lower surfaces of the patches. The electrodes between the patches and the host beam are grounded, the free electrodes of the two patches are first interconnected and then connected to a signal conditioning circuit, as shown in Figure 4D. To generate incident waves, an additional pair of patches is glued on the left side of the metabeam.

### Experimental Procedures

During the experiments of the metabeam in Figure 4D, both ends of the host beam are clamped. The TFs are created in Simulink and then encoded into dSPACE via a PC. To achieve time‐varying VRs, a repeating sequence block in Simulink is used to generate temporal parameters of the TFs. The sampling frequency for dSPACE is set at 40 kHz. An additional pair of patches are glued on the left side of the metabeam to generate incident waves (the excitation signal is generated by a dynamic signal analyzer (Brüel & Kjær PHOTON+) and amplified by a power amplifier (Krohn‐Hite Model 7602 M) by a factor of 7). For the experimental results in Figures 4 and 5 and Figures S18, S21, and S22 (Supporting Information), sinusoidal excitation signals are employed, and for the results in Figures S15, S16, and S23 (Supporting Information), broadband white noise signals are used. Amplitudes of the transmitted waves are measured by an accelerometer (Type 4516) mounted on the right side of the metabeam. In all the experiments, after ω_1,*j*_ are designed, ω_2,*j*_ are chosen according to $\omega_{2,j}={\left(\frac{1}{1-k_{31}^{2}}\right)}^{\frac{1}{2N}}\;\omega_{1,j}$.

### Numerical Simulations

The fully coupled 3D numerical simulations in frequency domain are performed in software COMSOL MULTIPHYSICS using the “Solid Mechanics,” “Electrostatics” and “Piezoelectric Effect” modules. For the simulations in time domain, first, a reduced model by using the mode superposition method is obtained; then, the reduced model and the circuits are modeled in the Simulink,^[^ ^43^ ^]^ a transport delay block is used to introduce a 28 µs time delay into the model.

### Time‐Varying Effective Constitutive Parameters of the Metabeam

Based on the classic Euler‐Bernoulli beam theory, and under the adiabatic and sub‐wavelength assumptions, the total bending stiffness of the unit cell shown in Figure 1A can be written as

(5) $$D_{\mathrm{tot}}=\frac{D_{A}D_{b}}{\left(1-\chi \right)D_{A}+\chi D_{b}}$$

in which, $D_{b}=E_{b}bh_{b}^{3}/12$ is the bending stiffness of the host beam, and *E*_b_, *b*,  *h*_b_ are the Young's modulus, width, and thickness of the host beam, respectively, χ  = *l*_p_/*l*_b_ is the covering ratio of the patch in the unit cell, *l*_p_, *l*_b_ are the length of the patch and the unit cell, respectively, *D*_A_ represents the bending stiffness of the middle sandwich part of the unit cell, the expression of it is

(6) $$D_{A}=D_{b}\;+E_{p}b\left[{\left(h_{b}+2h_{p}\right)}^{3}-h_{b}^{3}\right]/12$$

*E*_p_ is the effective Young's modulus of the shunted piezoelectric patch,

(7) $$E_{p}=E_{p}^{\mathrm{sh}}\;\frac{i\omega C_{p}^{s}/\left(1-k_{31}^{2}\right)+G}{i\omega C_{p}^{s}+G}$$

here, $E_{p}^{\mathrm{sh}}$ is the Young's modulus of a piezo‐patch under short‐circuit condition.

Substituting Equations (2), (6), and (7) into Equation (5), the total bending stiffness of the unit cell is written as a function of the parameters of TF

(8) $$D_{\mathrm{tot}}\;\left(t\right)=\lambda_{1}\frac{\left(\lambda_{2}-1\right)\prod_{j\;=\;1}^{N}\left(-\omega^{2}+i\beta_{j}\left(t\right)\omega_{1,j}\left(t\right)\omega +\omega_{1,j}^{2}\left(t\right)\right)-\lambda_{3}\prod_{j\;=\;1}^{N}\left(-\omega^{2}+i\beta_{j}\left(t\right)\omega_{2,j}\left(t\right)\omega +\omega_{2,j}^{2}\left(t\right)\right)}{\lambda_{2}\prod_{j\;=\;1}^{N}\left(-\omega^{2}+i\beta_{j}\left(t\right)\omega_{1,j}\left(t\right)\omega +\omega_{1,j}^{2}\left(t\right)\right)-\lambda_{3}\prod_{j\;=\;1}^{N}\left(-\omega^{2}+i\beta_{j}\left(t\right)\omega_{2,j}\left(t\right)\omega +\omega_{2,j}^{2}\left(t\right)\right)}$$

in which, λ_1_ = *D*_b_/(1 − χ) , λ_2_ = [1 + (1 + κ)(1 − χ)*D_r_*]/χ and λ_3_ = (1 − χ)*D*_r_/χ . $\kappa \;=1/(1-k_{31}^{2})$ and $D_{r}=E_{p}^{\mathrm{sh}}b\left[{\left(h_{b}+2h_{p}\right)}^{3}-h_{b}^{3}\right]/12D_{b}$.

The effective bending stiffness of the metabeam is defined as

(9) $$D_{\mathrm{eff}}\;\left(t\right)=\;\mathrm{Real}\left(D_{\mathrm{tot}}\left(t\right)\right)$$

Outside the bandgaps, the effective bending stiffness is positive, the effective loss factor can be directly defined as

(10) $$\eta_{\mathrm{eff}}\;\left(t\right)=\frac{I\mathrm{mag}\left(D_{\mathrm{tot}}\left(t\right)\right)}{D_{\mathrm{eff}}\left(t\right)}$$

Within the bandgaps, the effective bending stiffness becomes negative. The negative value of bending stiffness indicates that the material's response precedes excitation by π. To define the effective loss factor within the bandgaps, the absolute value of the bending stiffness is used to cancel the phase difference between the response and excitation caused by negative stiffness. Therefore, no matter within or outside the bandgaps, the effective loss factor can be defined as

(11) $$\eta_{\mathrm{eff}}\;\left(t\right)=\frac{I\mathrm{mag}\left(D_{tot}\left(t\right)\right)}{\left|D_{\mathrm{eff}}\left(t\right)\right|}$$

Parameters ω_1,*j*_, ω_2,*j*_, β_*j*_ in Equation (8) can be time‐dependent. Therefore, Equations (9) and (11) together describe a sort of temporal medium with inherent time‐varying constitutive parameters.

## Conflict of Interest

The authors declare no conflict of interest.

## Author Contributions

Conceptualization: K.Y.; Methodology: Z.L. and H.S.; Investigation: Z.L. and H.S; Experiment: Z.L.; Supervision: R.Z., X.Z., and G.H; Writing—original draft: K.Y., Z.L., and H.S.; Writing—review & editing: K.Y., Z.L., H.S., R.Z., X.Z., G.H., and G.H.

## Supporting information



Supporting Information

## Data Availability

The data that support the findings of this study are available from the corresponding author upon reasonable request.
