# Supplementary material for: Inherent Temporal Metamaterials with Unique Time‐Varying Stiffness and Damping
Source: Adv Sci (Weinh). 2024 Sep 25;11(43):2404695. doi: 10.1002/advs.202404695 (PMC11578320; doi:10.1002/advs.202404695)
Supplement: Supplementary file 1 — Supporting Information [file ADVS-11-2404695-s001.docx]

Supporting Information for

Inherent Temporal Metamaterials with Unique Time-Varying Stiffness and Damping

Zhiyuan Liu^1^, Kaijun Yi^1^*, Haopeng Sun^1^, Rui Zhu^1^, Xiaoming Zhou^1^, Gengkai Hu^1^,

Guoliang Huang^2^

*^1^School of Aerospace Engineering, Beijing Institute of Technology, Beijing 100081, People’s Republic of China.*

*^2^Department of Mechanics and Engineering Science, College of Engineering, Peking University, Beijing 100871, People’s Republic of China.*

**Email:* [*kaijun.yi@bit.edu.cn*](mailto:kaijun.yi@bit.edu.cn)

**This PDF file includes:**

Supplementary Note 1. Mimicking the resonant behavior of a 2-DOFs MR using the VR

Supplementary Note 2. The digital circuit used to run the VRs

Supplementary Note 3. The transfer function for simulating a VR connected to a piezo-patch

Supplementary Note 4. The effects of VR parameters on the effective constitutive parameters

Supplementary Note 5. Modulating the effective constitutive parameters in time domain

Supplementary Note 6. Method to calculate the scattering coefficients using the homogenized metamaterial beam model

Supplementary Note 7. Method to calculate the scattering coefficients using the fully coupled metamaterial beam model

Supplementary Note 8. Theoretical method to calculate the scattering coefficients using the effective bending stiffness

Supplementary Note 9. Corrections of the numerical models

Supplementary Note 10. Discussions on the maximum resonance frequency and modulation frequency of the proposed metabeam

Supplementary Note 11. The equivalent model of a 1D piezoelectric patch

Supplementary Note 12. Shaping the amplitudes and frequency spectra of waves in time domain

Supplementary Note 13. Geometry and material parameters of the fabricated metamaterial beam

Figures S1 to S23

Tables S1 and S2

**Supplementary Note 1. Mimicking the resonant behavior of a 2-DOFs MR using the VR**

Take the 2-DOFs MR in Figure 2A as an example, when the force is applied on the second mass, the mechanical addmitance is:

$$H\left(\omega\right)=\frac{X(\omega)}{F(\omega)}=\frac{1}{\left(-\omega^{2}m_{1}+i\omega c_{1}+k_{1}\right)-\frac{\left(i\omega c_{1}+k_{1}\right)^{2}}{-\omega^{2}m_{2}+i\omega\left(c_{1}+c_{2}\right)+\left(k_{1}+k_{2}\right)}}$$

The amplitude and phase of the above admittance are shown as the solid lines in Figure S1, the used parameters are: $m_{1}=m_{2}=1, c_{1}=c_{2}=2, k_{1}=k_{2}={10}^{6}.$

To mimic its behavior, a 2-poles TF is used, parameters of the TF is chosen as: $\omega_{1,1}=2\pi\times98.36$, $\omega_{1,2}=2\pi\times257.5$, $\omega_{2,1}=2\pi\times220.16$, $\omega_{2,2}=2\pi\times576.36$, $\beta_{1}=\beta_{2}=0.0015$, $\alpha=7.89\times{10}^{-11}$.

Amplitude and phase of the VR are also illustrated in Figure S1 through dashed lines. Apparently, the desinged VR has similar resonant behavior as the MR. Note that, the amplitudes of the 2-poles VR and the 2-DOFs MR are different at low frequencies near 0, and their phases differ by 90 degrees. These differences result from the multiplication of iω before the VR's transfer function, which is done to enhance stability.

**
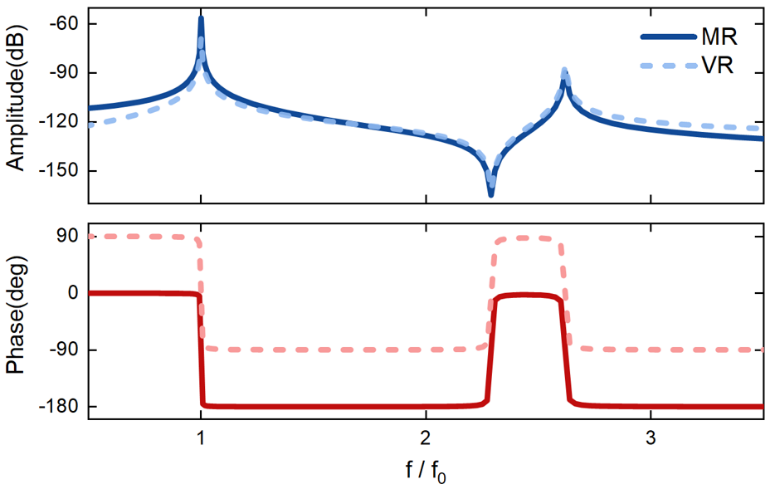
**

**Figure S1. Mimicking the resonant behavior of a 2-DOFs MR by the VR.** The upper panel and the lower panel are the amplitude-frequency curve and the phase-frequency curve, respectively. The solid lines and the dashed lines are the dynamic responses of MR and VR, respectively. $f_{0}$ is the first resonance frequency of MR.

**Supplementary Note 2. The digital circuit used to run the VRs**

The digital circuit contains a signal conditioning module and a controller (dSPACE is used in our studies). In the signal conditioning circuit shown in Figure S2A, the resistances $R_{1}$ and $R_{2}$ compose a voltage adaptor to adjust the input voltage signal to a safe range for the controller. The resistance $R_{3}$ and the operational amplifier serve as a current source driven by voltage and convert the controller’s output level into a current signal. The values of the resistance $R_{1}$, $R_{2}$ and $R_{3}$ are $4k\Omega$, $1k\Omega$ and $0.5k\Omega$, respectively. The dSPACE contains integrated ADC and DAC modules. The block diagram used to simulate the VR in the dSPACE is shown in Figure S2B. The gains $g_{1}$ and $g_{2}$ are set as 5 and 500 for offsetting the effects of voltage adaptor and current source in the signal conditioning circuit.

The schematic diagram of the experimental setup for verifying VRs is shown in Figure S2C. During the experiments, the VRs are encoded into dSPACE by PC. A dynamic signal analyzer (Brüel & Kjær PHOTON+) is employed to generate the broadband white noise voltage signal as the input into the digital circuit. Additionally, the analyzer also measures the output current signal from the digital circuit. The frequency response functions of VRs then can be calculated. In the experiment of testing VRs, after $\omega_{1,j}$ are designed, $\omega_{2,j}$ are given according to $1.05\omega_{1,j}$.


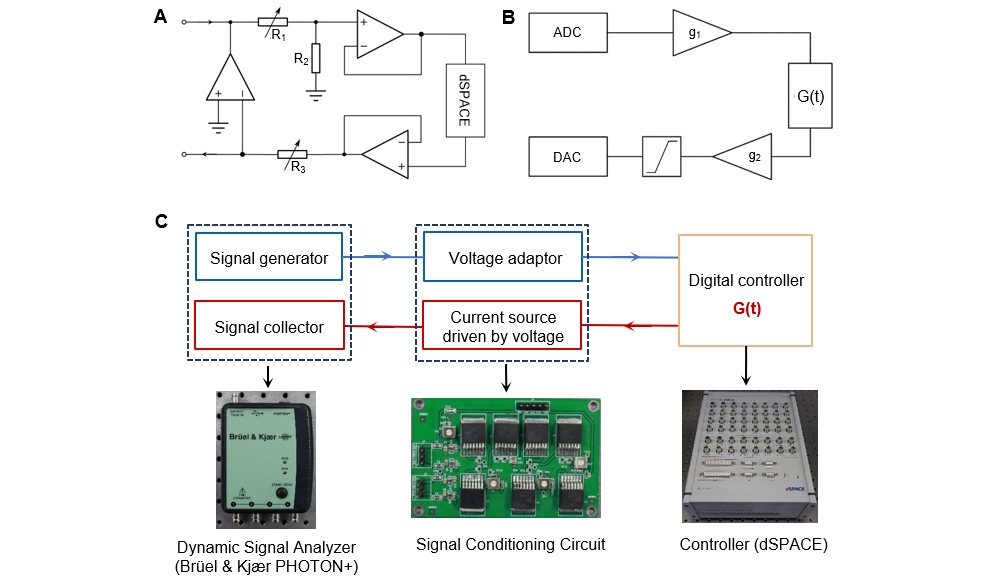


**Figure S2. The schematic diagrams for testing VRs.** (**A**) The schematic diagram of the digital circuit. (**B**) The processing blocks encoded in dSPACE. (**C**) The experimental setup for verifying VRs.

**Supplementary Note 3. The transfer function for simulating a VR connected to a piezo-patch**

We take the 1D piezoelectric patch shown in Figure S3 as an example. The polling direction of the piezoelectric material is direction 3. The patch is deformed in direction 1. The voltage between the upper and lower surfaces of the patch is *V*, the output current from the piezoelectric patch is *I*.


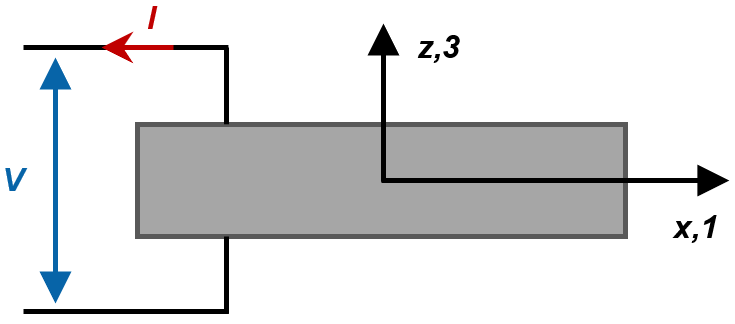


**Figure S3**. The schematic diagram of a 1D piezoelectric patch.

The 1D constitutive equations of piezoelectric material can be written as

$$S_{1}=S_{11}^{E}T_{1}+d_{31}E_{3}$$

$$D_{3}=d_{31}T_{1}+\varepsilon_{33}^{T}E_{3}$$

in which, $S_{1}$ and $T_{1}$ are the strain and stress, respectively; $E_{3}$ and $D_{3}$ are the electrical field and electrical displacement along the polling direction, respectively; $S_{11}^{E}$ is the compliance constant at constant electric field; $\varepsilon_{33}^{T}$ is the dielectric constant of the piezoelectric material at constant stress, and $d_{31}$ is the piezoelectric constant of the piezoelectric material.

Assuming that the whole electric field and electrical displacement on the upper and lower electrodes are uniform for the piezoelectric patch. The voltage and charge on surfaces of the piezoelectric patch can be obtained according to

$$V=-E_{3}h_{p}, Q=D_{3}A$$

in which, $h_{p}$ and *A* are the thickness and upper surface area of the piezoelectric patch.

The relation between strain, stress, voltage and charge of the piezoelectric patch can be derived as

$$T_{1}=E_{p}^{sh}S_{1}-\frac{E_{p}d_{31}}{h_{p}}V$$

$$Q=Ad_{31}E_{p}^{sh}S_{1}+C_{p}^{s}V$$

in which, $E_{p}^{sh}=1/{S_{11}^{E}}$ is in-plane Young’s modulus of the short-circuit (SC) piezoelectric patch; $k_{31}=d_{31}\sqrt{{E_{p}^{sh}}/{\varepsilon_{33}^{T}}}$ is the extensional coupling factor; $C_{p}^{s}={A\varepsilon_{33}^{T}\left( 1-k_{31}^{2} \right)}/{h_{p}}$ is the intrinsic capacitance of the patch at constant strain.

Considering the output current and the charge of piezoelectric patch have the relation $I=\dot{Q}$, Equation (S5) and Equation (S6) can be written as

$$V=\gamma_{1}S_{1}+\frac{1}{i\omega C_{p}^{s}}I$$

$$T_{1}=\gamma_{2}S_{1}+\frac{1}{i\omega\gamma_{3}}I$$

in which, $\gamma_{1}=\frac{Ad_{31}E_{p}^{sh}}{C_{p}^{s}}$, $\gamma_{2}=\frac{E_{p}^{sh}}{{1-k}_{31}^{2}}$ and $\gamma_{3}=\frac{{h_{p}C}_{p}^{s}}{d_{31}E_{p}^{sh}}$.

Equation (S7) suggests that a deformed piezo-patch is equivalent to a voltage source $V_{s}=\gamma_{1}S_{1}$ plus a capacitor $C_{p}^{s}$ in series. Due to the presence of this intrinsic capacitance, the TF $G^{*}$ for simulating a VR connected to a patch should be modified. The total admittance of the TF $G^{*}$ and the intrinsic capacitance $C_{p}^{s}$ should equal to the admittance of the original TF *G*, which can be expressed as

$$G=\left(\frac{1}{G^{*}}+\frac{1}{i\omega C_{p}^{s}}\right)^{-1}$$

The TF for simulating a VR connected to a patch then can be deduced as Equation (2).

**Supplementary Note 4. The effects of VR parameters on the effective constitutive parameters**

We take a VR with one resonance as an example to analyze the effects of VR’s parameters on the effective constitutive parameters. For a one-pole VR, there are two parameters in its TF, namely, $\omega_{1,1}$ and $\beta_{1}$. The impact of parameter $\omega_{1,1}$​ on the effective bending stiffness and loss factor is illustrated in Figure S4A. This parameter can adjust the location of significant changes in the effective constitutive parameters. Figure S4B demonstrates the effects of parameter $\beta_{1}$ on the effective constitutive parameters. When $\beta_{1}$ is relatively small, the resonating strength is strong, allowing the effective bending stiffness to vary from positive to negative regions. As $\beta_{1}$ increases, the resonating strength weakens, and the range of effective bending stiffness variation becomes narrower. When $\beta_{1}$ is set to a relatively large value, the effective bending stiffness can only be modulated within the positive region, and the extremums of the loss factor are eliminated. Thus, $\beta_{1}$ influences the resonating strength of the VR and adjusts the variation range of the constitutive parameters around the resonance frequency.


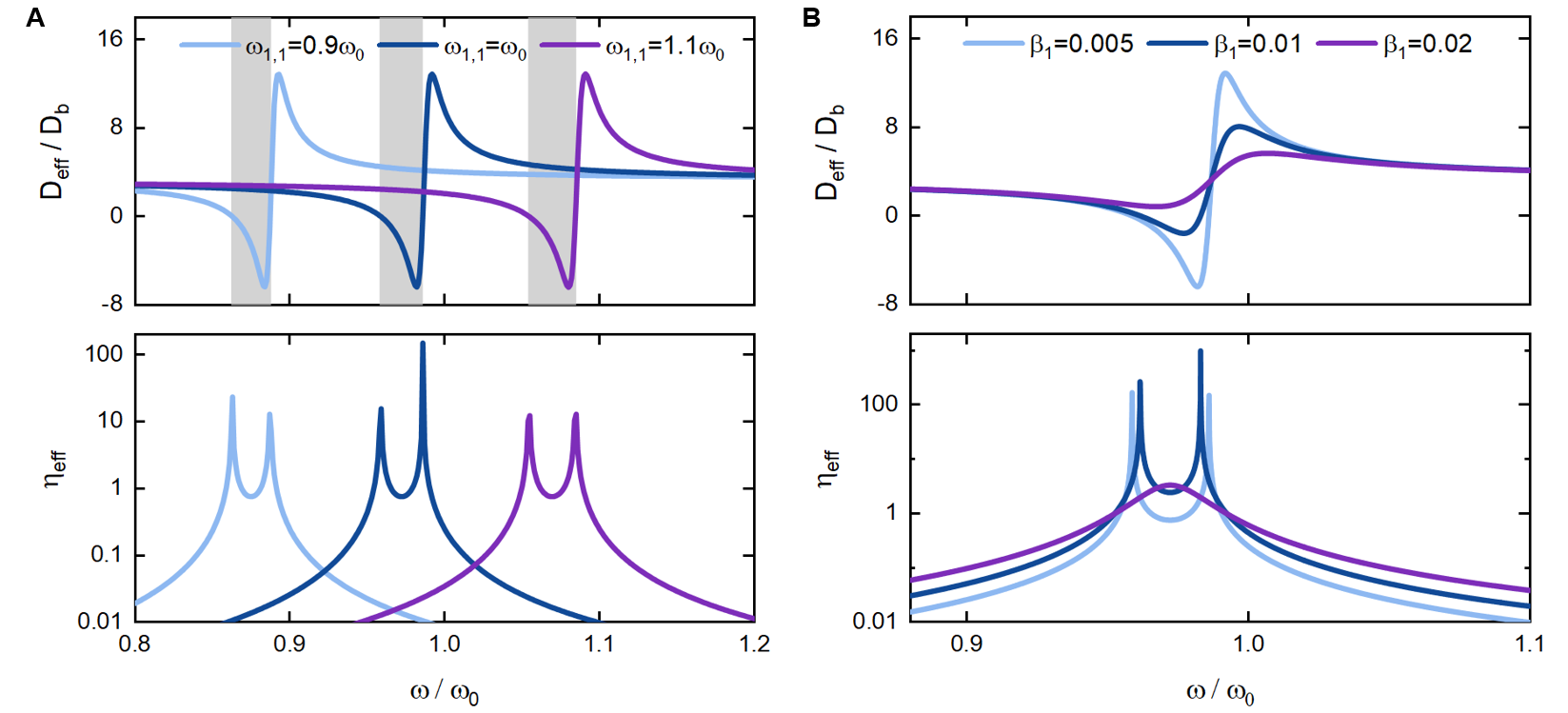


**Figure S4. The effects of VR’s parameters (A)** $\boldsymbol{\omega}_{\mathbf{1,1}}$ **and (B) $\boldsymbol{\beta}_{\mathbf{1}}$ on the effective bending stiffness and loss factor. $\boldsymbol{\omega}_{\mathbf{0}}$ is a targeting frequency.**

**Supplementary Note 5. Modulating the effective constitutive parameters in time domain**

In Figure 3 we show that the effective constitutive parameters can be modulated in time domain by tuning the resonating strength. Here, we show that they can also be time-modulated by changing the resonance frequency. We use a one-pole TF to do the simulations, the results are shown in Figure S5. We periodically tune parameter $\omega_{1,1}$ in the time domain (see Figure S5A), the resultant time-varying effective bending stiffness and loss factor are illustrated in Figure S5 (B and C), respectively. The constitutive parameters at frequencies within the varying range of the resonance frequency $\omega_{1,1}$ (see Figure S5 (D and E)) are dramatically modulated in the time domain.


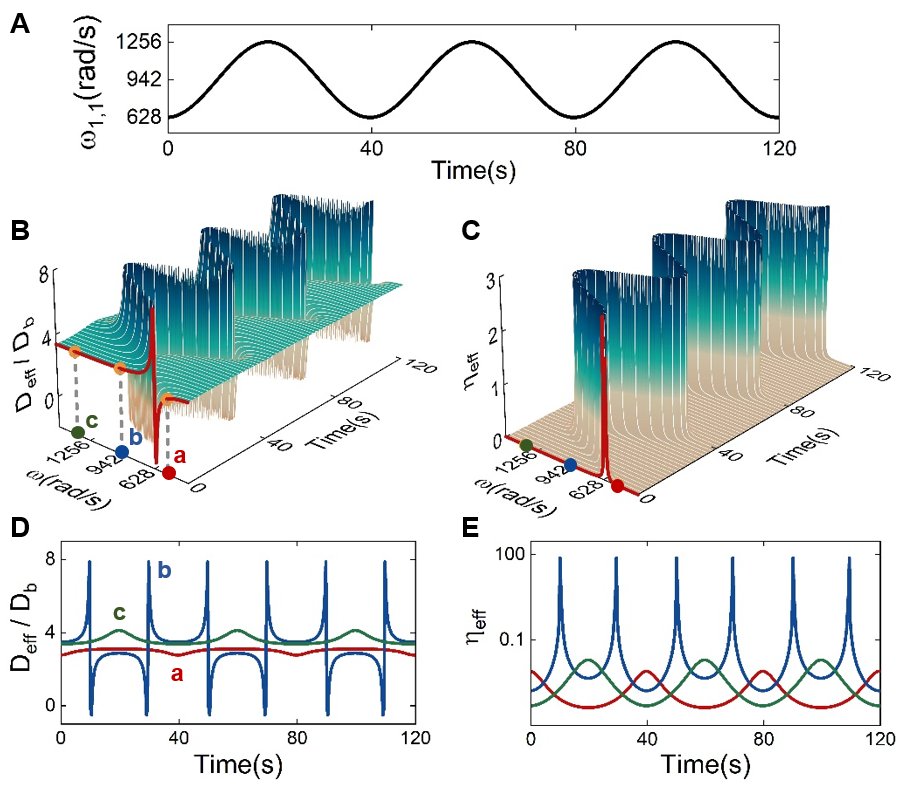


**Figure S5**. **Effective constitutive parameters time-modulated by varying $\boldsymbol{\omega}_{\boldsymbol{1,1}}$ of a one-pole TF.** (**A**) Time-varying pattern of parameter $\omega_{1,1}$. (**B** and **C**) The time-varying effective bending stiffness and loss factor of the metamaterial beam induced by the temporal $\omega_{1,1}$. (**D** and **E**) The time-varying constitutive parameters at frequencies a, b and c. Frequency b is within the varying range of resonance frequency $\omega_{1,1}$, frequencies a, c are outside this range.

Furthermore, we can modulate parameter $\omega_{1,1}$ and $\beta_{1}$ of the one-pole TF in time domain simultaneously (see Figure S6A) to obtain time-varying effective bending stiffness (see Figure S6B) and loss factor (see Figure S6C).


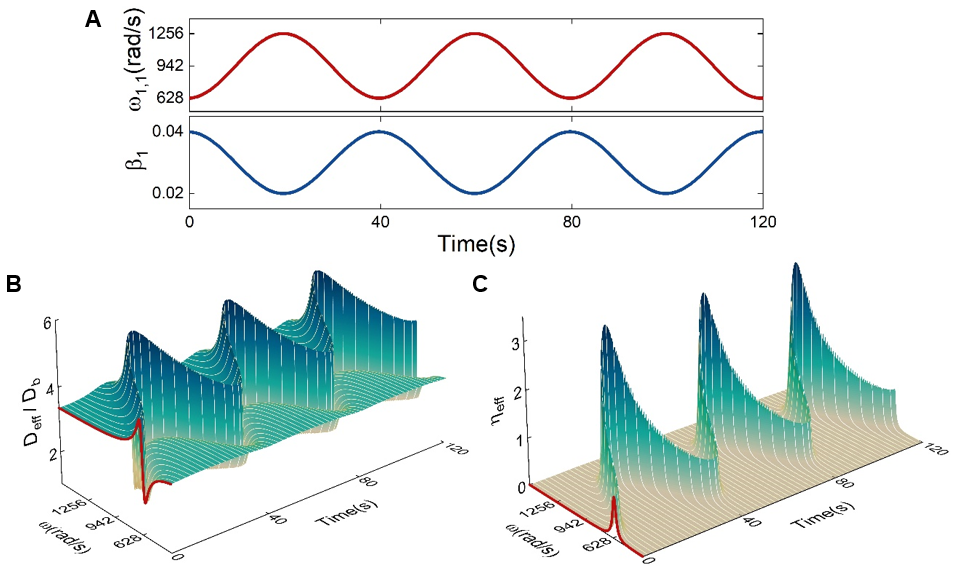


**Figure S6**. **Effective constitutive parameters time-modulated by varying $\boldsymbol{\omega}_{\boldsymbol{1,1}}$ and $\boldsymbol{\beta}_{\boldsymbol{1}}$ of a one-pole TF simultaneously**. (**A**) The upper panel and lower panel are the time-varying pattern of parameter $\omega_{1,1}$ and $\beta_{1}$ of the one-pole TF, respectively. (**B** and **C**) The time-varying effective bending stiffness and loss factor of the metamaterial beam induced by the temporal $\omega_{1,1}$ and $\beta_{1}$.

Besides, we can dramatically modulate the constitutive parameters within several frequency bands at the same time by utilizing multi-pole TFs. Without loss of generality, a three-pole TF is used for demonstration. We modulate $\beta_{1,j=1,2,3}$ with different amplitude or phase in the time domain (see Figure S7A). The resultant time-varying effective bending stiffness and loss factor are illustrated in Figure S7B and Figure S7C, respectively. It is observed that the effective constitutive parameters near the 3 resonance frequencies $\omega_{1,j=1,2,3}$ are dramatically modulated in time domain.


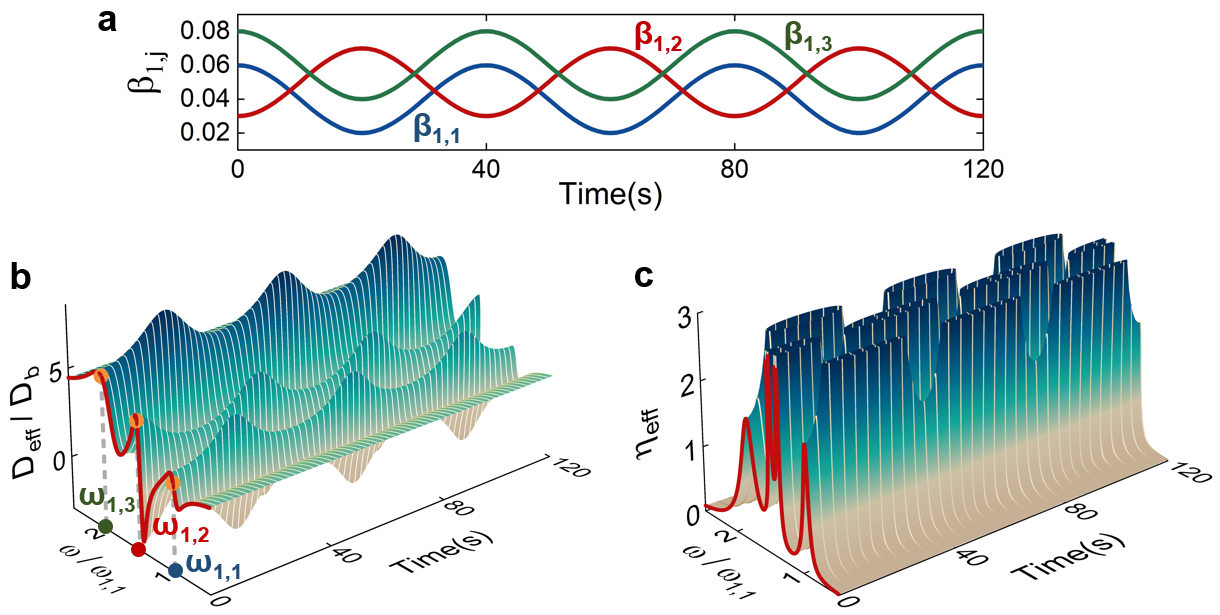


**Figure S7**. **Effective constitutive parameters time-modulated by a three-pole TF**. (**A**) The time-varying patterns of $\beta_{1,j=1,2,3}$. (**B** and **C**) The time-varying effective bending stiffness and loss factor of the metamaterial beam induced by the temporal $\beta_{1,j=1,2,3}$.

**Supplementary Note 6. Method to calculate the scattering coefficients using the homogenized metamaterial beam model**

The schematic diagram of the model for calculating the reflection, absorption and transmission coefficients of the homogenized metamaterial beam is shown in Figure S8. The model is a single beam consisting of two parts marked with different colors. The grey parts are made of aluminum. The blue part is the homogenized metamaterial beam, the effective Young’s modulus and density of it is given as

$$E_{eff}=12D_{tot}/bh_{b}^{3}$$

$$\rho_{eff}=m_{tot}/bh_{b}$$

in which, $m_{tot}$ is the total linear mass of a unit cell and can be expressed as

$$m_{tot}=\chi m_{A}+\left(1-\chi\right)m_{b}$$

here, $m_{A}=\rho_{b}bh_{b}+2\rho_{p}bh_{p}$ and $m_{b}=\rho_{b}bh_{b}$. The expression of $D_{tot}$ is given in Equation (8).

The simulations are done in software COMSOL MULTIPHYSICS. Port boundary conditions are applied on both sides of the beam to excite and absorb elastic waves. Here, we only consider the flexural wave mode and define the left and right boundary as Numeric port 1 and Numeric port 2, respectively. In the study, boundary mode analysis is firstly performed on each port to compute the propagating flexural wave mode, which can be obtained by searching around $k_{f}=\sqrt[4]{{12\rho_{b}\omega_{1,1}^{2}}/{Y_{b}h_{b}^{2}}}$, $\omega_{1,1}$ is the angular frequency of the incident wave. Then, we activate port sweep to excite an incident flexural wave at port 1 in the frequency domain. The scattering parameters $S_{21}$ and $S_{11}$ corresponding to the transmission and reflection coefficients can be automatically calculated, and the absorption coefficient can be defined as $A_{b}=\sqrt{1-S_{11}^{2}-S_{21}^{2}}$. The results of the scattering properties calculated using the homogeneous model with effective constitutive parameters are shown as the solid lines depicted in Figure 4C.


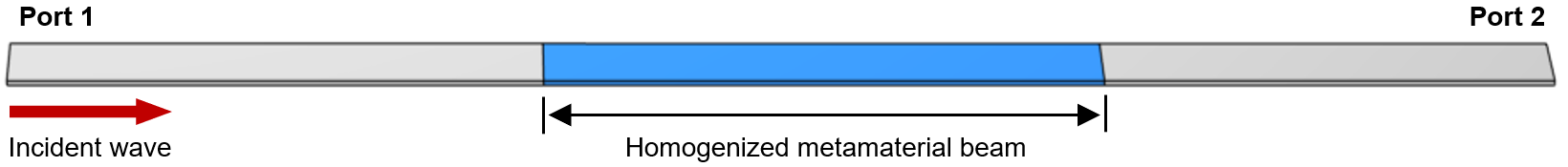


**Figure S8. The model used to calculate reflection, absorption and transmission coefficients of the homogenized metamaterial beam.**

We also use the homogenized metamaterial beam to calculate amplitudes of the transmitted waves when both ends of the beam are clamped (see Figure S9). A pair of piezo-patches is applied on the left side of the homogenized metamaterial beam for excitation, the acceleration at a point on the right side is measured. Since we work in the adiabatic time-varying regime, at each moment, the metamaterial beam can be regarded as a static beam, the corresponding transmitted waves can be calculated using static analysis. By manually updating the constitutive parameters of the homogenized metamaterial beam, we can obtain the time-varying patterns of the transmitted waves.


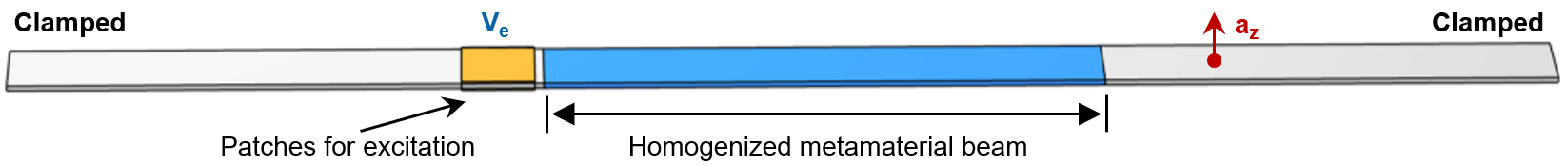


**Figure S9. The model for calculating transmission waves through the homogenized metamaterial beam.**

**Supplementary Note 7. Method to calculate the scattering coefficients using the fully coupled metamaterial beam model**

The reflection, absorption and transmission coefficients of waves incident on the metamaterial beam are also calculated using an electromechanically coupled model. As shown in Figure S10, the model consists of a metamaterial beam with 7 unit cells and two uniform beams (Aluminum) on both sides of the metamaterial beam. The TFs for simulating the VRs connected to the patches are incorporated into the model using weak contribution boundary conditions. Port boundary conditions are applied on both sides of the whole beam. By using the method introduced in Supplementary Note 6, the scattering coefficients of the fully coupled metamaterial beam can be calculated and shown as the dot lines depicted in Figure 4C.


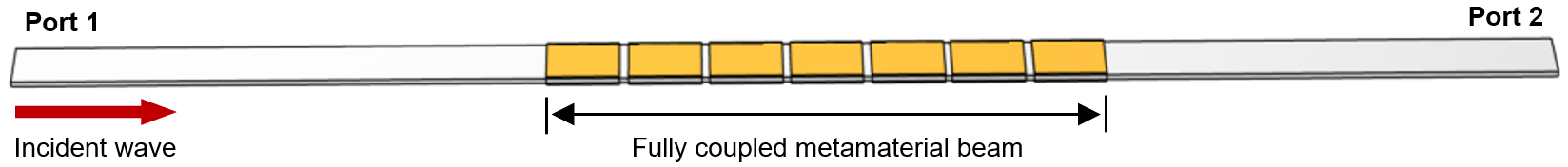


**Figure S10. The model used to calculate reflection, absorption and transmission coefficients of the fully coupled metamaterial beam.**

**Supplementary Note 8. Theoretical method to calculate the scattering coefficients using the effective bending stiffness**

The transfer matrix method can be used to calculate the scattering coefficients of flexural waves in beam with multiple layers and interfaces shown in Figure S11.


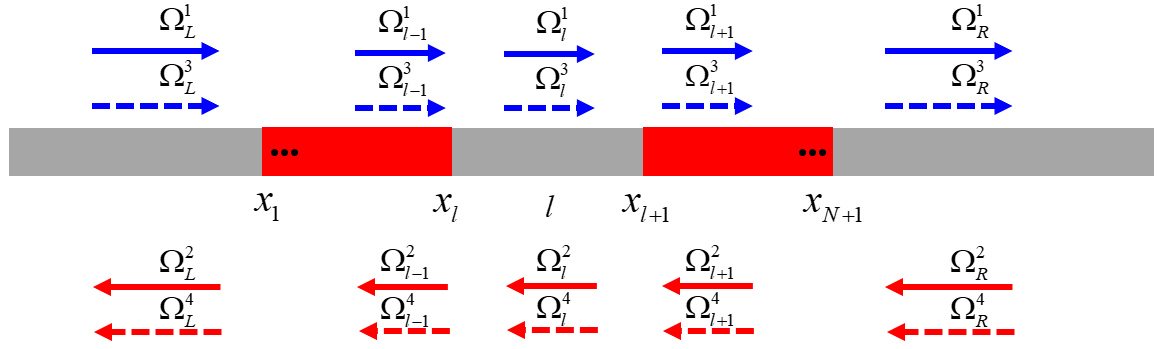


**Figure S11. Schematically showing the multiple layers and the interfaces of a beam.** The solid lines and dashed lines are the propagating and evanescent flexural waves, respectively.

The equation governing flexural waves in beam is

$$D\frac{\partial^{4}W}{\partial x^{4}}+\rho\frac{\partial^{2}W}{\partial t^{2}}=0$$

where *W* is the displacement in the vertical direction, *D*, *ρ* are the bending stiffness and the linear density of the beam. The solutions of Equation (S13) consist of evanescent and propagating flexural waves. Therefore, the displacement field in region *l* can be written as

$$W_{l}\left(x\right)=\Omega_{l}^{1}e^{ik_{l}x}+\Omega_{l}^{2}e^{-ik_{l}x}+\Omega_{l}^{3}e^{-k_{l}x}+\Omega_{l}^{4}e^{k_{l}x}$$

in which, $k_{l}$ is the wave number in layer *l*. The displacement field can also be expressed as a column vector $W_{l}\left( x_{l} \right)=\left[ W_{l} {dW_{l}}/{dx}{D_{l}d^{2}W_{l}}/{dx^{2} {D_{l}d^{3}W_{l}}/{dx^{3}}} \right]^{T}$. $D_{l}$is the bending stiffness of the beam in layer *l.* The displacement field at $x_{l}$ in the layer *l* can be further expressed as

$$W_{l}\left(x_{l}\right)=M_{l}^{l}\left(\begin{matrix} \Omega_{l}^{1} \\ \Omega_{l}^{2} \\ \Omega_{l}^{3} \\ \Omega_{l}^{4} \end{matrix}\right)$$

in which

$$M_{l}^{l}=\left(\begin{matrix} (ik_{l})^{0}e^{ik_{l}x_{l}} & (-ik_{l})^{0}e^{-ik_{l}x_{l}} & (-k_{l})^{0}e^{-k_{l}x_{l}} & (k_{l})^{0}e^{k_{l}x_{l}} \\ (ik_{l})^{1}e^{ik_{l}x_{l}} & (-ik_{l})^{1}e^{-ik_{l}x_{l}} & (-k_{l})^{1}e^{-k_{l}x_{l}} & (k_{l})^{1}e^{k_{l}x_{l}} \\ D_{l}(ik_{l})^{2}e^{ik_{l}x_{l}} & D_{l}(-ik_{l})^{2}e^{-ik_{l}x_{l}} & D_{l}(-k_{l})^{2}e^{-k_{l}x_{l}} & D_{l}(k_{l})^{2}e^{k_{l}x_{l}} \\ D_{l}(ik_{l})^{3}e^{ik_{l}x_{l}} & D_{l}(-ik_{l})^{3}e^{-ik_{l}x_{l}} & D_{l}(-k_{l})^{3}e^{-k_{l}x_{l}} & D_{l}(k_{l})^{3}e^{k_{l}x_{l}} \end{matrix}\right)$$

Hence, it is straightforward to derive

$$W_{l}\left(x_{l+1}\right)=M_{l}^{l+1}\left(M_{l}^{l}\right)^{-1}W_{l}\left(x_{l}\right)$$

where the associated transfer matrix of layer *l* is $\Pi_{l}=M_{l}^{l+1}\left( M_{l}^{l} \right)^{-1}$, According to the displacement continuity condition, the interfaces of layer *l* have the relationship

$$W_{l}\left(x_{l+1}\right)=W_{l+1}\left(x_{l+1}\right)$$

Therefore, if there are *N* layers, the total transfer matrix is $\Pi_{s}=\Pi_{N}\cdot\Pi_{N-1}\cdots\Pi_{l}\cdots\Pi_{1}$. If we define also the incoming and outgoing matrices in the same way, the overall transfer matrix of the multilayered structure can be derived as

$$M^{s}=\left(M_{out}^{N+1}\right)^{-1}\Pi_{s}M_{in}^{1}$$

The relationship between the incoming waves and the outgoing waves of the multilayered beam in Figure S11 is

$$M^{s}\left(\begin{matrix} \Omega_{L}^{1} \\ \Omega_{L}^{2} \\ \Omega_{L}^{3} \\ \Omega_{L}^{4} \end{matrix}\right)=\left(\begin{matrix} \Omega_{R}^{1} \\ \Omega_{R}^{2} \\ \Omega_{R}^{3} \\ \Omega_{R}^{4} \end{matrix}\right)$$

By rearranging the terms that correspond to incoming and outgoing flexural waves, the Equation (S20) can be derived as

$$\left(\begin{matrix} M_{11}^{s} & M_{13}^{s} & 0 & 0 \\ M_{2}^{s} & M_{23}^{s} & -1 & 0 \\ M_{31}^{s} & M_{33}^{s} & 0 & 0 \\ M_{41}^{s} & M_{43}^{s} & 0 & -1 \end{matrix}\right)\left(\begin{matrix} \Omega_{L}^{1} \\ \Omega_{L}^{3} \\ \Omega_{R}^{2} \\ \Omega_{R}^{2} \end{matrix}\right)=\left(\begin{matrix} -M_{12}^{s} & -M_{14}^{s} & 1 & 0 \\ -M_{22}^{s} & -M_{24}^{s} & 0 & 0 \\ -M_{32}^{s} & -M_{34}^{s} & 0 & 1 \\ -M_{42}^{s} & -M_{44}^{s} & 0 & 0 \end{matrix}\right)\left(\begin{matrix} \Omega_{L}^{2} \\ \Omega_{L}^{4} \\ \Omega_{R}^{1} \\ \Omega_{R}^{3} \end{matrix}\right)$$

Without loss of generality, we excite the system from the left side of the beam, which makes $\Omega_{L}^{1}=0$ and $\Omega_{L}^{3}=\Omega_{R}^{2}=\Omega_{R}^{4}=0$. By this definition, we have the reflection (transmission) coefficients $r_{1}$ and $r_{2}$ ($t_{1}$ and $t_{2}$) of the propagating and evanescent waves, respectively. This linear system can be written as

$$\left(\begin{matrix} -M_{12}^{s} & -M_{14}^{s} & 1 & 0 \\ -M_{22}^{s} & -M_{24}^{s} & 0 & 0 \\ -M_{32}^{s} & -M_{34}^{s} & 0 & 1 \\ -M_{42}^{s} & -M_{44}^{s} & 0 & 0 \end{matrix}\right)^{-1}\left(\begin{matrix} M_{11}^{s} & M_{13}^{s} & 0 & 0 \\ M_{2}^{s} & M_{23}^{s} & -1 & 0 \\ M_{31}^{s} & M_{33}^{s} & 0 & 0 \\ M_{41}^{s} & M_{43}^{s} & 0 & -1 \end{matrix}\right)\left(\begin{matrix} 1 \\ 0 \\ 0 \\ 0 \end{matrix}\right)=\left(\begin{matrix} r_{1} \\ r_{2} \\ t_{1} \\ t_{2} \end{matrix}\right)$$

with the scattering matrix expressed as

$$S=\left(\begin{matrix} -M_{12}^{s} & -M_{14}^{s} & 1 & 0 \\ -M_{22}^{s} & -M_{24}^{s} & 0 & 0 \\ -M_{32}^{s} & -M_{34}^{s} & 0 & 1 \\ -M_{42}^{s} & -M_{44}^{s} & 0 & 0 \end{matrix}\right)^{-1}\left(\begin{matrix} M_{11}^{s} & M_{13}^{s} & 0 & 0 \\ M_{2}^{s} & M_{23}^{s} & -1 & 0 \\ M_{31}^{s} & M_{33}^{s} & 0 & 0 \\ M_{41}^{s} & M_{43}^{s} & 0 & -1 \end{matrix}\right)$$

The reflection and transmission coefficients of the propagating waves then can be obtained：

$$r_{1}=S_{11}=\frac{\left|\begin{matrix} -M_{21}^{s} & M_{24}^{s} \\ -M_{41}^{s} & M_{44}^{s} \end{matrix}\right|}{\left|\begin{matrix} M_{22}^{s} & M_{24}^{s} \\ M_{42}^{s} & M_{44}^{s} \end{matrix}\right|}, t_{1}=S_{31}=M_{11}^{s}+M_{12}^{s}S_{11}+M_{14}^{s}S_{21}$$

According to the above transfer matrix method, the bending stiffness appearing in the transfer matrix is a key parameter for adjusting the scattering coefficients of the beam.

**Supplementary Note 9. Corrections of the numerical models**

Due to the time delay in the digital circuit and the manufacturing error, there are non-negligible differences between the numerical and experimental models. To quantitatively validate the experimental results, we have made reasonable corrections to the numerical models.

First, the error caused by time delay is corrected. The time delay is induced by both the signal conditioning circuit and the digital controller, it causes a phase lag between the output and input signals. Using this phase lag, we can experimentally measure the actual time delay of a system. To measure the time delay of our digital circuit, we encode the TF $G=1/{100}$ into the digital controller. We send a broadband white noise signal into the circuit and measure the output. The phase lag between the output and input signals is calculated and shown in Figure S12. The time delay therefore can be obtained according to $\tau=\left| \Theta\left( \omega\right) \right|/\omega$, $\Theta\left( \omega\right)$ is the phase lag at frequency $\omega$. The measured time delay of our circuits is about 28 $\mu s$ and is independent on the frequency.


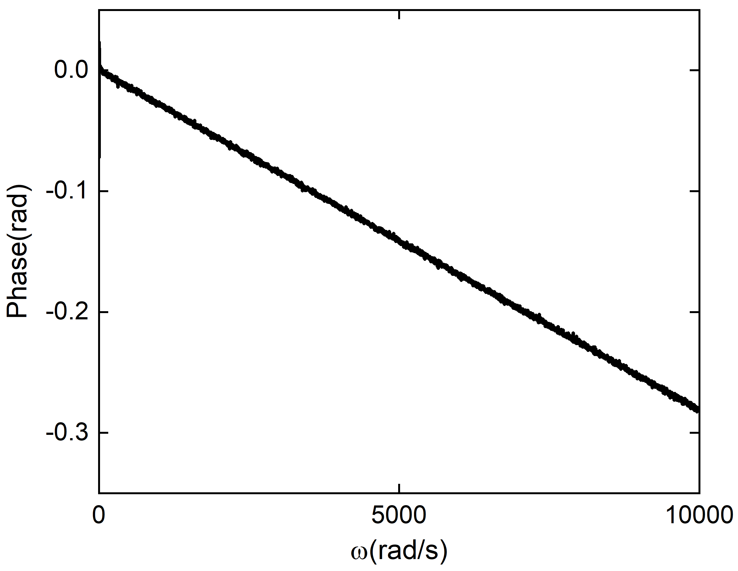


**Figure S12. The phase lag between the output and input signals of the digital circuit when the TF $\boldsymbol{G=}\boldsymbol{1}/\boldsymbol{100}$ is used.**

The 28 $\mu s$ time-delay is considered in our numerical simulations. Figure S13 shows the caculated and measured frequency response (FR) curves of a three-pole VR with resonance frequencies at 500Hz, 1000Hz and 1500Hz. Very good agreement between the numerical and experimental results are obtained, which further verifies that the delay of the digital circuit is 28 $\mu s$.


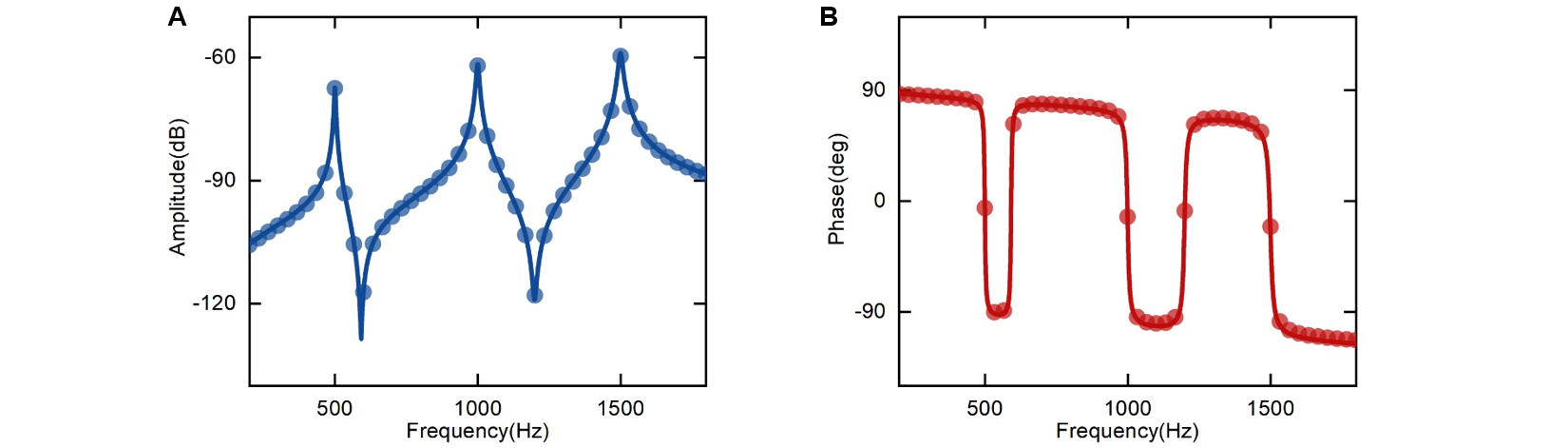


**Figure S13. The amplitude-frequency characteristic curves (A) and phase-frequency characteristic curves (B) of a three-pole TF.** The solid lines and dot lines are the experimental results and numerical results, respectively.

Note that, in the time-domain simulations of the metamaterial beam, the time delay is considered by adding a transport delay block in the Simulink model (see Method); in the frequency-domain simulations, we take the time delay into consideration in another way. The time delay introduces a negative damping into the metamaterial beam. Due to this negative damping, $\beta_{j}$ in Equation (2) cannot be too small, otherwise, the whole system will be unstable. To offset the negative damping caused by time delay, an extra $\beta_{extra\_j}$ is used to bring in positive damping in the experiments. To determine this value, we gradually increase $\beta_{j}$ from zero until the experimental system becomes stable, this value is confirmed as the extra value needed to offset the intrinsic negative damping. In the frequency-domain simulations, this extra $\beta_{extra\_j}$ value is added to the $\beta_{j}$ value used in the theoretical analysis to take the time delay into consideration. We also note that in Figures 4, 5 and in the rest of this Supplementary material, the presented $\beta_{j}$ values are the theoretical ones when these is no time delay.

Second, the error caused by manufacturing is corrected. In this process, the patches of the metamaterial beam are open-circuited (OC). The fully coupled model in Figure S14 is used. We excite on the left side of the metamaterial beam and measure the response at a point on the right side, the transmission curve therefore can be numerically or experimentally obtained. We adjust the thickness of the beam in the numerical model until the numerical result is coincident with the experimental one. When the thickness of the beam is modified to 2.8 mm (the original value is 3 mm), the numerical and experimental results have acceptable agreements, as shown in Figure S15.


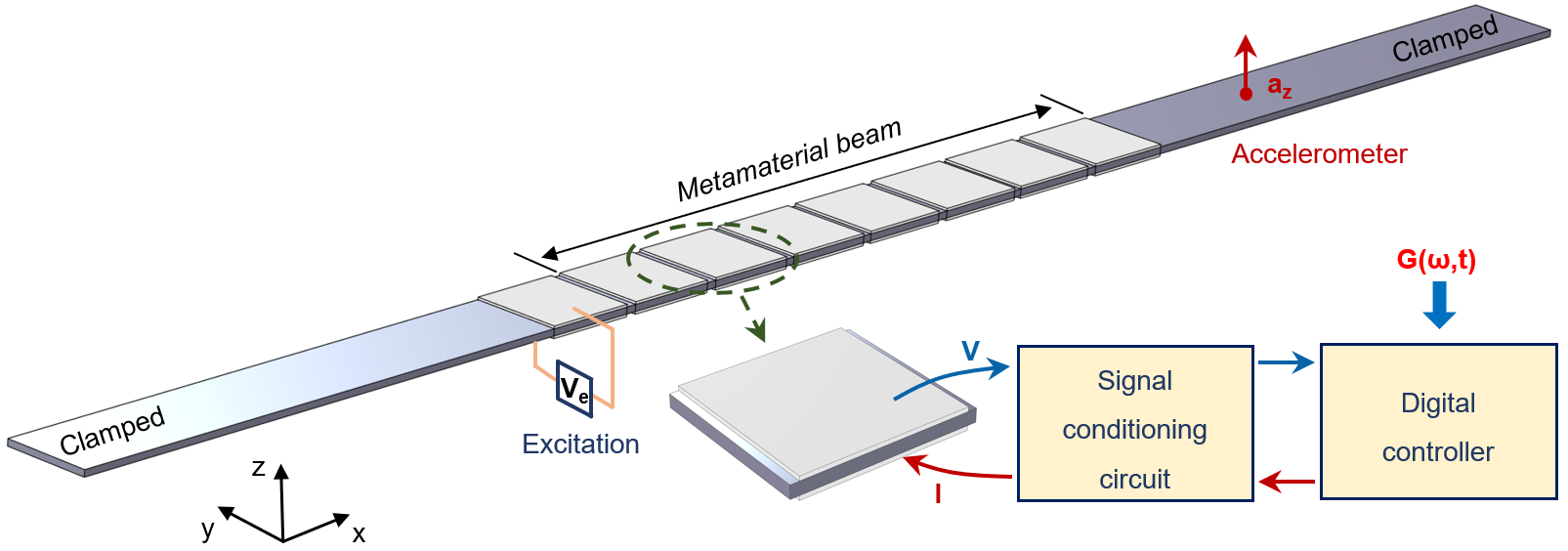


**Figure S14. The sketch of the fully coupled numerical model.**


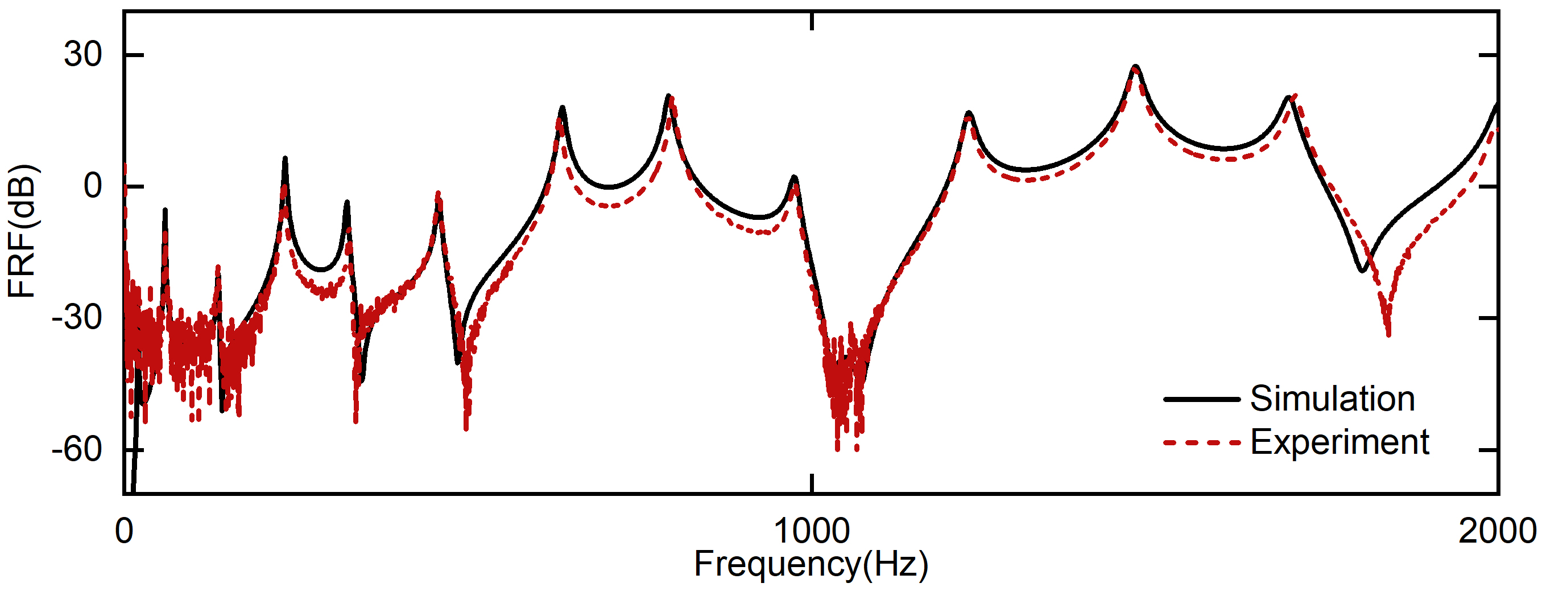


**Figure S15. Numerically and experimentally obtained transmittance curves of the metamaterial beam when patches are open-circuited.** The black solid line and red dashed line are numerical and experimental results, respectively.

After the above corrections made to the homogenized and fully coupled metamaterial beam models, now, we compare the experimental and numerical results to check the accuracy of these models. A one pole TF is involved, the resonance frequency is fixed at $\omega_{1,1}(=2\pi*633)$, three different $\beta_{1}$ values are used, they are 0.05, 0.3 and 0.6. Figure S16 compares the numerical and experimental transmittance curves in different cases. It is observed that they have good agreements with each other with little discrepancies been noticed. In Figure S16 (B to D), when VRs are involved, the measured maximum amplitudes are higher than simulated results, these differences may be caused by the electric damping of the real circuits, which is not considered in the numerical models. Due to the above factor, the experimentally measured transmitted waves (in Figure 4 (H to J), Figure 5 (A to D, F and G) and Figure S16) have a little bit larger amplitudes than those from the numerical simulations.


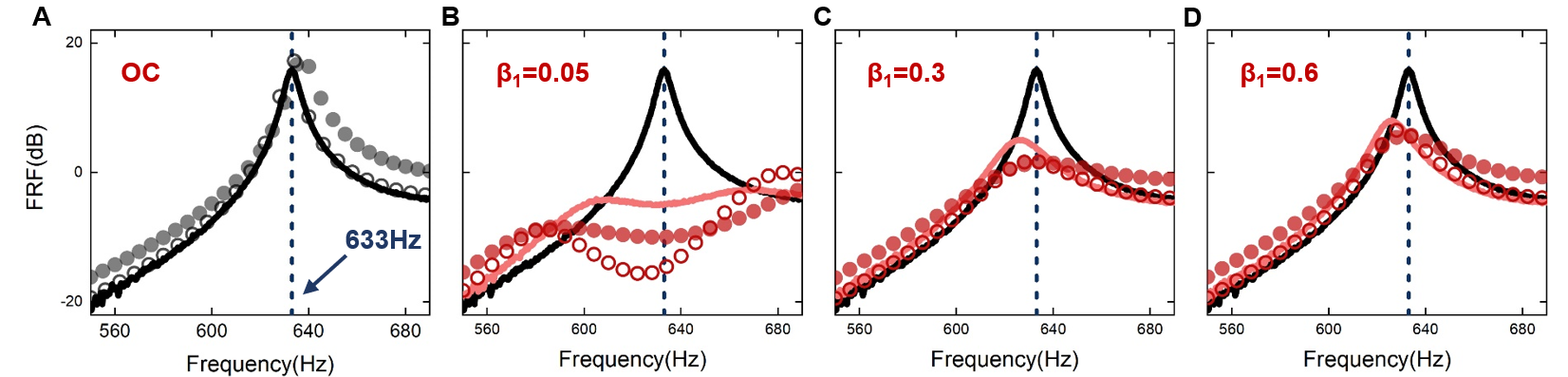


**Figure S16. The transmittance curves of the metamaterial beam when piezo-patches are open-circuited (A) or connected with VRs with parameter (B) $\boldsymbol{\beta}_{\boldsymbol{1}}\boldsymbol{=0.05}$, (C) $\boldsymbol{\beta}_{\boldsymbol{1}}\boldsymbol{=0.3}$ and (D) $\boldsymbol{\beta}_{\boldsymbol{1}}\boldsymbol{=0.6}$.** The solid lines are the results of experiments, the hollow and filled circle lines are the numerical results of the homogenized model and fully coupled model, respectively. The black and red lines indicate the transmittance curves when piezo-patches are open-circuited and connected with VRs, respectively. The blue dashed lines indicate the resonance frequency (633 Hz) of the VRs.

**Supplementary Note 10. Discussions on the maximum resonance frequency and modulation frequency of the proposed metabeam**

The maximum resonance frequency and modulation frequency mainly determined by the geometry size of the beam as well as the working frequencies of the electronics used in the digital circuits. If we assume that the electronics can perfectly operate at all frequencies, the maximum resonance frequency is limited by the length of the unit cell of the metabeam, because we have assumed that the wavelength of the flexural wave in the beam is much longer than the length of one unit cell. To find out the maximum resonance frequency of the proposed metabeam under the assumption aforementioned, we make a comparison of the transmission and the reflection coefficients between the fully coupled model and the homogenized model when the resonance frequency of VR is 650Hz (Figure S17 (A and B)), 1500Hz (Figure S17 (C and D)) and 2500 Hz (Figure S17 (E and F)), respectively. With the increase of the resonance frequency, the differences of the scattering coefficients near the resonance obtained from the fully coupled model and the homogenized model become significant. The homogenized model is insufficient to describe the wave transmission effects when the resonance frequency is up to 2500Hz, which means the sub-wavelength assumption is not suitable when the resonance frequency is sufficiently high. To determine the maximum frequency for which homogenization remains valid, we analyzed the transmission and reflection waves of the metabeam with the piezo-patches open-circuited (Figure S17 (G and H)). It is evident that beyond 1500 Hz, there is a substantial discrepancy between the results from the homogenized model and those from the numerical model. Consequently, the resonance frequency of the VR must be less than 1500 Hz. Reducing the length of the unit cell can increase the maximum resonance frequency.


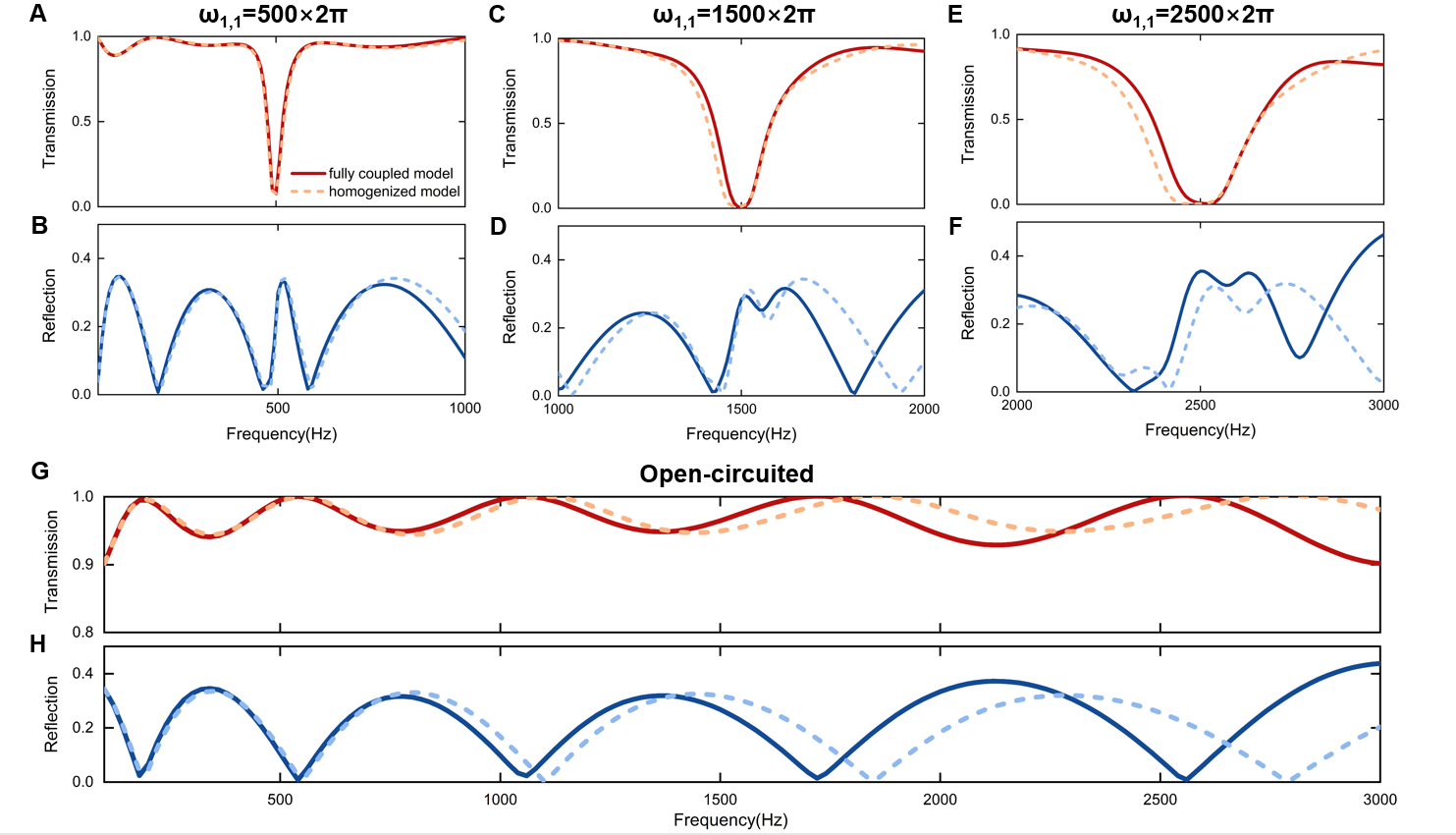


**Figure S17. The transimission and reflection coefficients of the metabeam when the resonance frequency of VR is 650 Hz (A and B), 1500 Hz (C and D) , 2500 Hz (E and F) or the piezo-patches are open-circuited (G and H), repectively.** The solid lines and dashed lines are the results obtained by the fully coupled numerical model and the homogenized beam model, respectively.

According to the results in Figure 5(A to D), we conclude that the modulation frequency should be less than 0.1 time of the resonance frequency. To further verify this conclusion, we change the resonance frequency to 975Hz and excite the incident waves at the same frequency. The transmitted waves through the metabeam when modulation frequencies are 30Hz, 60Hz, 90Hz and 120Hz are shown in Figure S18 (A to D), respectively. When the modulation frequency is less than 90Hz (about 0.1 times of the resonance frequency), the amplitudes of the transmitted waves in the simulations approximately follow the patterns predicted by the homogenized model. However, when the modulation frequency is up to 120Hz, the homogenized model is no longer suitable to describe the dynamic responses of it.


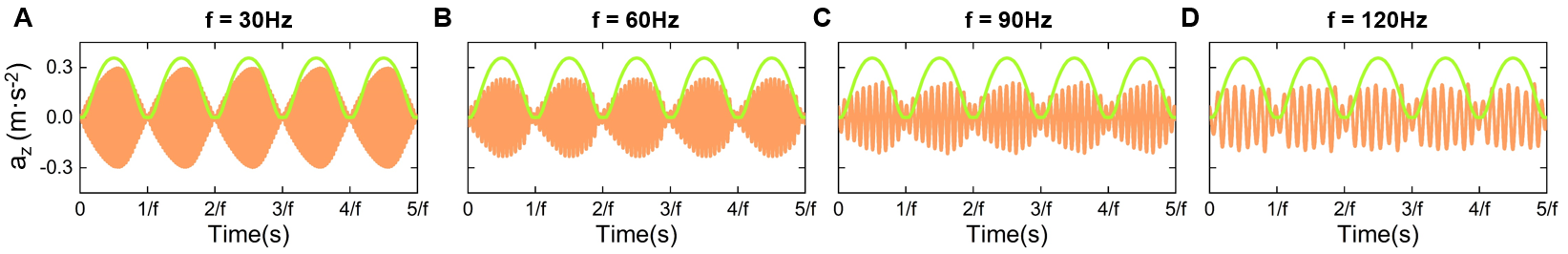


**Figure S18. Variation of the trasmitted waves through the metabeam when the modulation frequency is 30 Hz (A), 60 Hz (B), 90 Hz (C) and 120 Hz (D), respectively.** The resonance frequency of the metabeam and the frequency of incident wave are all 975 Hz. The orange lines are the results of numerical simulations. The green solid lines are the results obtained using theoretical effective constitutive parameters.

**Supplementary Note 11. The equivalent model of a 1D piezoelectric patch**

A 1D piezoelectric patch under longitudinal expansion is shown in Figure S4. The polarization direction of the piezoelectric patch is along the thickness direction 3, which is vertical to the expansion direction 1 when the metabeam is under longitudinal motion.


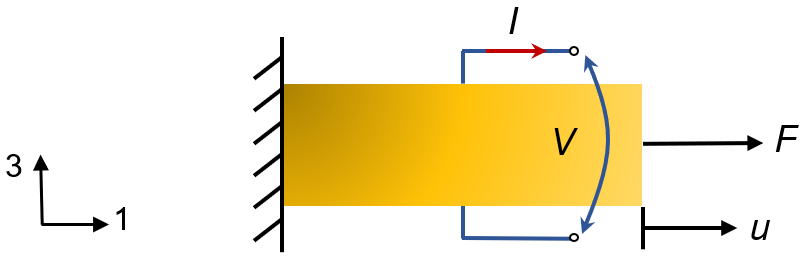


**Figure S19. Schematic of a 1D piezoelectric patch under longitudinal motion.**

According to Equations (S2) and (S3), the 1D constitutive equations of the piezoelectric patch can be further expressed as

$$\begin{matrix} T_{1}=C_{11}^{E}S_{1}-e_{31}E_{3} \\ D_{3}=e_{31}S_{1}+\varepsilon_{33}^{s}E_{3} \end{matrix}$$

in which, $C_{11}^{E}$ is the stiffness constant at constant electric field; $\varepsilon_{33}^{s}$ is the dielectric constant of the piezoelectric material at constant strain, and $e_{31}$ is the piezoelectric constant of the pieoelectric patch.

The integration of Equation. (S25) over the volume of the patch (of length $l_{p}$ and cross-section *A*) leads to

$$\begin{matrix} F=k_{p}u+\gamma V \\ Q=\gamma u-C_{p}^{s}V \end{matrix}$$

where the variables

$$F=T_{1}A, u=S_{1}l_{p}, V=-E_{3}l_{p}, Q=D_{3}A$$

represent the force acting on the patch, its deformation, the voltage across its electrodes and the charge flowing through them, respectivly. The constants

$$k_{p}=\frac{C_{11}^{E}A}{l_{p}}, \gamma=\frac{e_{31}A}{l_{p}}, C_{p}^{s}=\frac{\varepsilon_{33}^{s}A}{l_{p}}$$

are the short-circuited stiffness, piezoelectric coupling constant and capacitance at constant strain of the patch, respectively.

Considering the charge, output current and the voltage of piezoelectric patch have the relations $I=\dot{Q}$ and $I=G^{*}V$. The Equation (S26) can be deduced as

$$F=k_{p}u+\gamma^{2}\frac{s}{G^{*}+sC_{p}^{s}}u$$

in which, $G^{*}$ is the equivalent admittance of the shunt circuit.

The equivalent mechanical model of the 1D piezoelectric patch shunted with one-pole VR is shown in Figure S20. The equilibrium equation of the model is

$$F=k_{p}u+\tan^{2}\theta\left(-m\omega^{2}+i\omega c+k\right)u$$

According to Equations (S29) and (S30), it is evident that the electromechanical coupling phenomenon exhibited by the piezo-patch can be analogously depicted by two hinged, inclined rigid bars. Specifically, the magnitude of the electromechanical coupling factor, denoted as *γ*, corresponds precisely to the tangent of the angle *θ*, where *θ* represents the angle formed between the vertical axis and the inclined rigid bar.


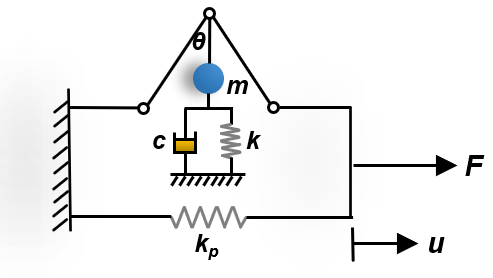


**Figure S20. The equivalent mechanical model of the 1D piezoelectric patch.**

**Supplementary Note 12. Shaping the amplitudes and frequency spectra of waves in time domain**

As an additional example, we utilize the metabeam as an elastic Morse coder to demonstrate its capability in modulating wave amplitudes in an aperiodic manner.

Morse codes represent letters of the alphabet, numerals and punctuation marks by an arrangement of dots, dashes and spaces, as shown in Figure S21A. Traditionally, the codes are transmitted as electric pulse, mechanical or visual signals. Here, we program the codes into the constitutive parameters of the metabeam. For this end, we define three states by using a one-pole TF (resonates at 633 Hz) with three different $\beta_{1}$ values to mimic the dash, dot and space used in traditional Morse code, respectively. These states are distinguished by the amplitudes of the transmitted waves when the metabeam is excited on the left side by a constant sine signal at 633 Hz. Specifically, a “dash” is obtained by setting $\beta_{1}$ to 0.2, the measured transmitted wave has the largest amplitude among the three states and it is normalized to unit 1, as shown in Figure S21B; to represent a dot, $\beta_{1}=0.1$ is used, the normalized amplitude of the transmitted wave is reduced to 0.5 (Figure S21C); a space between letters is linked to $\beta_{1}=0$, the amplitude of the transmitted wave is approximate 0 (Figure S21D); in addition, each state is designed to last 2 seconds in time domain. Using the above coding rules, we encrypt “BIT”, which is the abbreviation of *Beijing Institute of Technology*, into the metabeam by using a time-variant TF, $\beta_{1}$ of this TF and the induced time-varying constitutive parameters are shown in Figure S21 (E to G). The encoded information can be extracted by mechanically stimulating the metabeam on the left-side through a constant sine signal at 633 Hz and measuring the transmitted wave on the right-side, the measured signal is shown in Figure S20H, which clearly transmits the letters “BIT”.


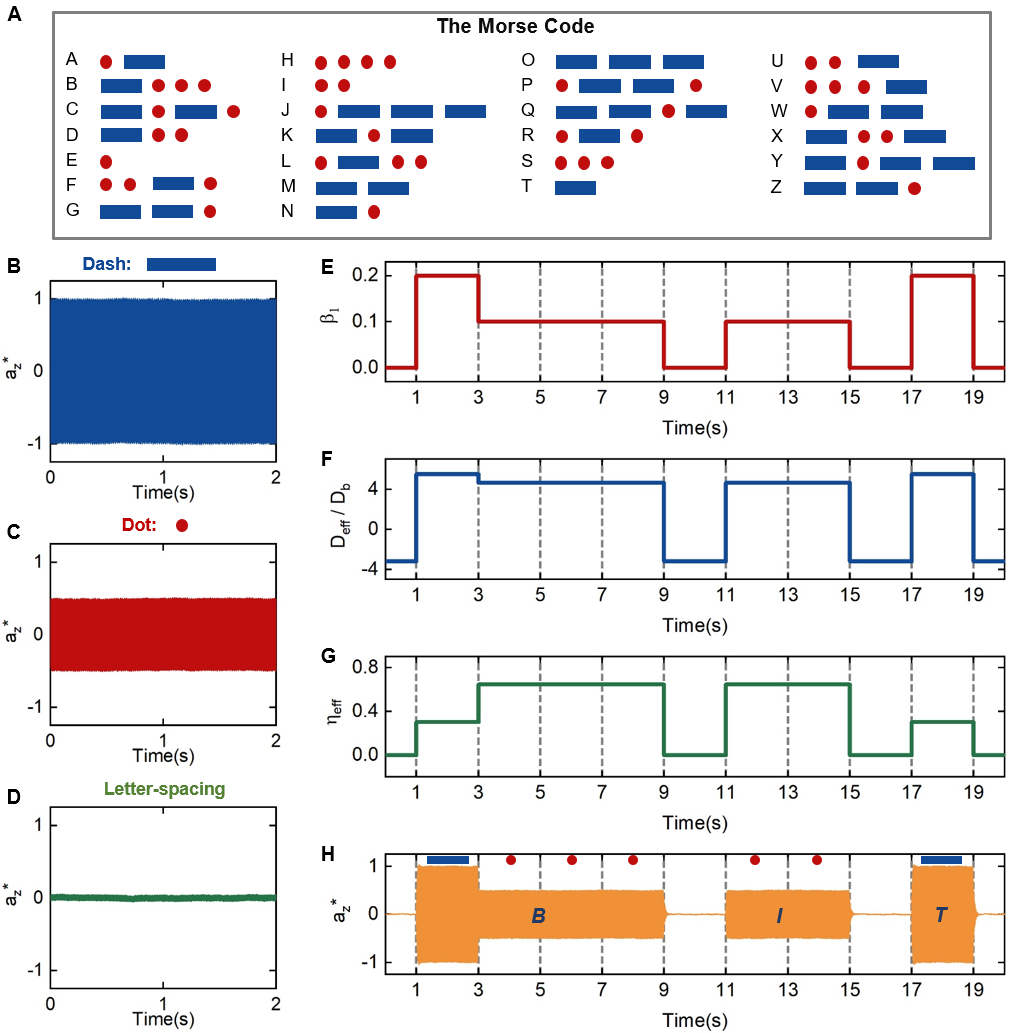


**Figure S21. An elastic Morse coder realized using the temporal metabeam.** (**A**) The Morse code table. (**B** to **D**) Three states are defined by using a one-pole TF (the resonance frequency is 633 Hz) with three different $\beta_{1}$ values to mimic the “dash” (**B**), “dot” (**C**) and “space” (**D**) used in traditional Morse code, respectively. These states are distinguished by the amplitudes of the transmitted waves when the metabeam is excited on the left side by a sine signal at 633 Hz. The “dash” is obtained by setting $\beta_{1}$ to 0.2, the measured transmitted wave has the largest amplitude among the three states and it is normalized to unit 1; a “dot” is obtained by using $\beta_{1}$= 0.1, the normalized amplitude of the transmitted wave is reduced to 0.5; a space between letters is linked to $\beta_{1}$= 0, the amplitude of the transmitted wave is approximate 0; in addition, each state is designed to last 2 seconds in time domain. $a_{z}^{*}$ means the normalized amplitude. (**E**) Time-domain variation pattern of the parameter $\beta_{1}$ of the TF used to write the message “BIT”, which is the abbreviation of *Beijing Institute of Technology*, into the metabeam. (**F** and **G**) The induced time-varying bending stiffness and loss factor at 633 Hz. (**H**) The encoded information in the metabeam is extracted by mechanically stimulating the metabeam on the left-side through a sine signal at 633 Hz and measuring the transmitted wave on the right-side. The measured transmitted signals carry the message “BIT” according to the Morse code table in Figure S21A.

According to the Morse code table, we can use the metamaterial beam to generate all the 26 letters, the results are shown in Figure S22.


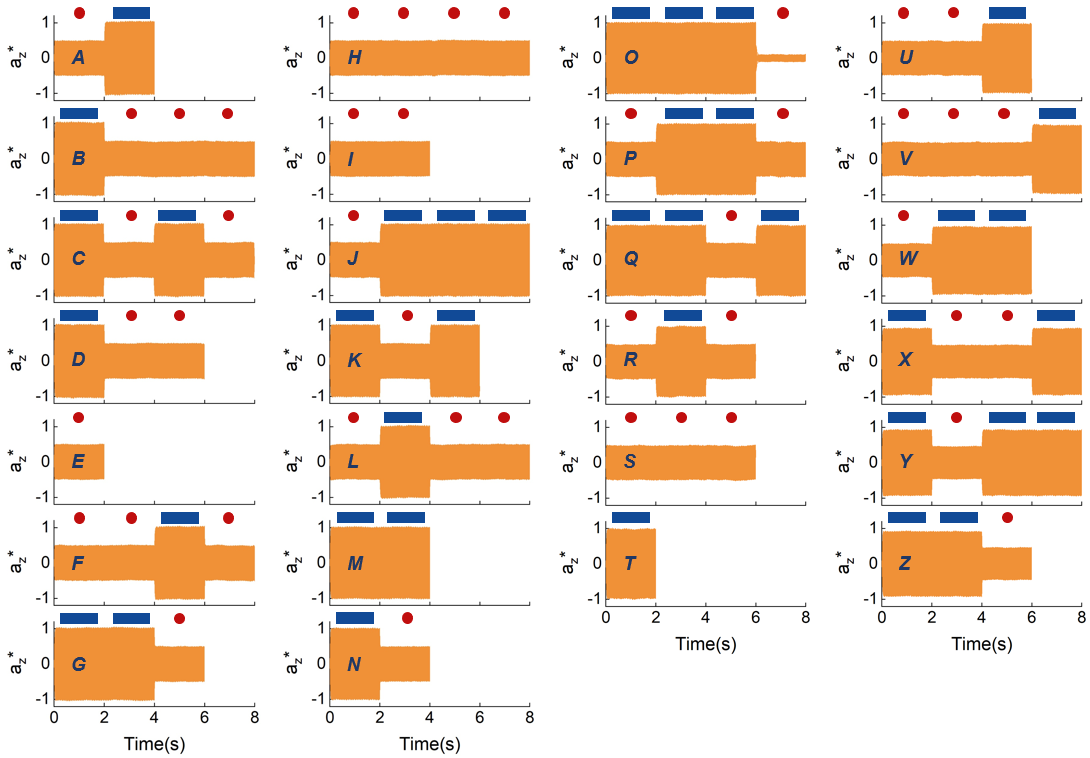


**Figure S22. All 26 letters generated by using the elastic Morse coder.**

Furthermore, to showcase the spectrum shaping capability of the metabeam, we have implemented a frequency-hopping filter.

A frequency-hopping filter can automatically change its stopbands in a designed pattern. To achieve such functionality, we use a TF with temporally varying resonance frequencies to simulate the VR. The resonance frequencies of the VR are programmed to vary in time domain in a fashion shown in Figure S23A. For $t=$0-5, 5-10 and 10-15 seconds, the resonance frequency is 640 Hz, 805 Hz and 982 Hz, respectively. For $t=$15-20 seconds, the VR simultaneously resonates at 640 Hz, 805 Hz and 982 Hz. To verify the frequency-hopping effects, waves with broadband spectrum are excited on the left side of the metamaterial beam, the transmitted waves to the right side are measured, as shown in Figure S23B. We perform FFT operation to the measured signals between $t=$0-5, 5-10, 10-15 and 15-20 seconds, respectively, results are illustrated in Figure S23 (C to F). It is clearly observed that the structural filter’s working frequency jumps from 640Hz to 805Hz and then becomes 982Hz, after t=15s, the filter works simultaneously at these three frequencies.


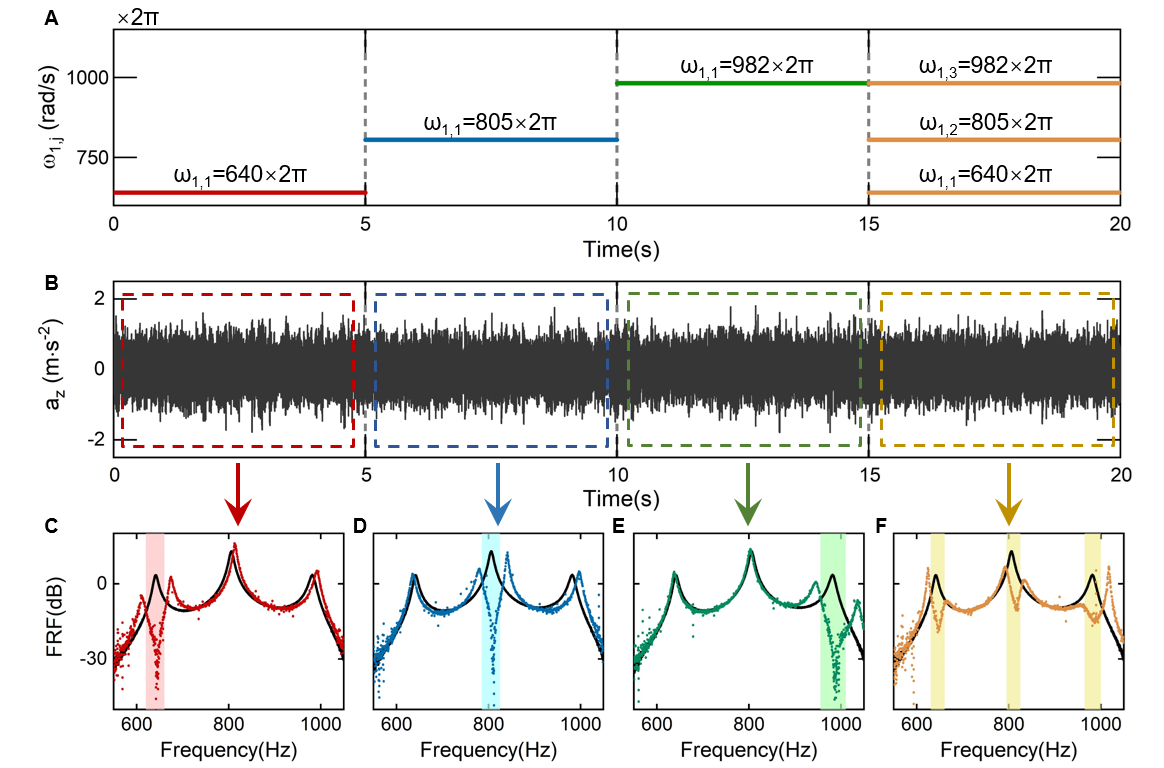


**Figure S23. A frequency-hopping structural filter realized by exploiting the time-varying characteristics of the metabeam.** (A) The resonance frequencies of the VR varying in time domain. For t=0~5, 5~10 and 10~15 seconds, the VR has a single resonance frequency equal to 640 Hz, 805 Hz and 982 Hz, respectively. For t=15~20 seconds, the VR simultaneously resonates at 640 Hz, 805 Hz and 982 Hz. (B) The temporal transverse acceleration signal measured on the right side of the metabeam when waves with broadband spectrum are excited on the left side. (C to F) The spectra of the measured acceleration signal between different time intervals. The structural filter’s working frequency jumps from 640 Hz to 805 Hz and then becomes 982 Hz, at last, the filter works at these three frequencies simultaneously.

**Supplementary Note 13. Geometry and material parameters of the fabricated metamaterial beam**

The geometry and material parameters of one unit cell of the fabricated metamaterial beam in Figure 4D are presented in Table S1. The intrinsic capacitances of the patches are measured using a Precision LCR Meter (TH2838H) and the values are listed in Table S2.

Table S1. Geometry and material parameters of one unit cell

| Parameters | Values | Values |
| --- | --- | --- |
| Material | Aluminum | PZT-8 |
| Length | $\text{l}_{\text{b}}\text{=42mm}$ | $\text{l}_{\text{p}}\text{=38mm}$ |
| Width | $\text{w}_{\text{b}}\text{=38.1mm}$ | $\text{w}_{\text{p}}\text{=38.1mm}$ |
| Thickness | $\text{h}_{\text{b}}\text{=3mm}$ | $\text{h}_{\text{p}}\text{=1mm}$ |
| Density | $\text{ρ}_{\text{b}}\text{=2700kg∙}\text{m}^{\text{-3}}$ | $\text{ρ}_{\text{b}}\text{=7700kg∙}\text{m}^{\text{-3}}$ |
| Young’s modulus | $\text{Y}_{\text{b}}\text{=70GPa}$ | $\text{Y}_{\text{p}}\text{=79.4GPa}$ |
| Coupling constant | **——** | $\text{d}_{31}\text{=-1.14e10C/N}$ |
| Relative permittivity under constant stress | **——** | $\text{ε}_{3}^{\text{σ}}\text{=990}$ |

**Table S2. The measured intrinsic capacitances of the patches in all unit cells**

|  | 1 | 2 | 3 | 4 | 5 | 6 | 7 |
| --- | --- | --- | --- | --- | --- | --- | --- |
| Capacitance | 25.36nF | 25.65nF | 25.53nF | 26.35nF | 26.64nF | 26.54nF | 26.21nF |
